# STAT6 Activation Exacerbates Ferroptosis in Airway Epithelium by Inhibiting PRKN‐Mediated Mitophagy in Pulmonary Fibrosis

**DOI:** 10.1002/advs.202501718

**Published:** 2025-07-17

**Authors:** Youjing Yang, Guangbin Huang, Tao Zhang, Yi Ling, Junyu Jiang, Dingyuan Du, Yu Ma, Shasha Tao

**Affiliations:** ^1^ Chongqing Key laboratory of Emergency Medicine Chongqing University Central Hospital Chongqing Emergency Medical Center Chongqing 400014 China; ^2^ Department of Trauma Surgery Chongqing University Central Hospital Chongqing Emergency Medical Center Chongqing 400014 China; ^3^ School of Medicine Soochow University Suzhou 215006 China

**Keywords:** ferroptosis, mitophagy, pulmonary fibrosis, rifabutin, STAT6

## Abstract

Pulmonary fibrosis (PF) remains a clinically intractable condition with limited therapies. Ferroptosis has emerged as a critical driver of PF. The previous study demonstrates that increased secretion of tissue plasminogen activator from ferroptotic airway epithelial cells contributes to PF progression in a paracrine manner. Herein, the indispensable role of signal transducer and activator of transduction 6 (STAT6) is further elucidated in maintaining airway epithelial homeostasis during PF and uncovers a novel mechanism by which STAT6 regulates mitophagy to modulate ferroptosis. Specifically, mitophagy is induced during PF along with STAT6 activation, and deficiency of STAT6 significantly alleviates epithelial ferroptosis and PF. Mechanistically, STAT6 directly binds to the parkin RBR E3 ubiquitin protein ligase (PRKN) promoter region at the site (−990 to −976), inhibiting PRKN transcription and thereby impairing mitophagy. Consistently, lentivirus‐mediated PRKN interference in both wild‐type and STAT6 knockout mice aggravates ferroptosis and PF. Furthermore, virtual screening identifies rifabutin as a potential STAT6 inhibitor, that exhibits therapeutic effects against PF both in vivo and in vitro. Collectively, these findings reveal an unreported mechanism by which STAT6 promotes PF by inhibiting PRKN‐mediated mitophagy in the airway epithelium. Rifabutin is further identified and validated as a promising STAT6 inhibitor to alleviate PF, offering new insights into therapy development.

## Graphical abstract

This study uncovers STAT6‐driven pulmonary fibrosis (PF) via suppression of PRKN‐mediated airway epithelial mitophagy, triggering mitochondrial dysfunction and ferroptosis. Rifabutin is identified as a potent STAT6 inhibitor that effectively reverses fibrotic progression. These findings reveal the STAT6‐PRKN axis as a pathogenic regulator and provide a mechanistically grounded therapeutic strategy, bridging mechanistic discovery to translational innovation in PF treatment.

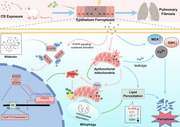

## 1 Introduction

Pulmonary fibrosis (PF) is a fatal fibrotic lung disease characterized by progressive and irreversible scarring of lung tissue, culminating in respiratory failure.^[^ ^1^, ^2^, ^3^ ^]^ Over the past few decades, there has been a marked increase in the prevalence and incidence of PF, which poses major social and economic challenges.^[^ ^4^, ^5^, ^6^ ^]^ Despite the clinical approval of two antifibrotic drugs, nintedanib and pirfenidone, these treatments merely decelerate the decline in pulmonary function without arresting the fibrotic process.^[^ ^7^, ^8^ ^]^ Consequently, elucidating the molecular mechanisms underlying PF is imperative for developing novel therapeutic strategies to enhance patient prognosis and survival rates.

The pathogenesis of PF is believed to be initiated by airway epithelial injury, which occurs within a dysregulated inflammatory microenvironment and impaired wound healing processes.^[^ ^9^, ^10^, ^11^ ^]^ The dysfunction of epithelial progenitor cells and persistent epithelial damage are central contributors to fibrogenesis. Among these factors, iron metabolism has garnered increasing attention because of its dual role in maintaining physiological functions and contributing to pathological conditions.^[^ ^12^, ^13^ ^]^ Emerging evidence has implicated disrupted iron homeostasis in the pathogenesis of PF. For instance, histological analyses of patients with idiopathic pulmonary fibrosis (IPF) revealed extracellular iron deposition and macrophage hemosiderin accumulation.^[^ ^14^ ^]^ Similarly, studies using bleomycin‐induced PF models linked abnormal mitochondrial iron metabolism to disease progression.^[^ ^15^ ^]^ Iron overload in Hfe‐deficient mice further corroborated the role of iron dysregulation in fibrosis.^[^ ^16^ ^]^ Our previous study highlighted the pivotal role of ferroptosis, a regulated form of cell death driven by iron‐dependent lipid peroxidation, in PF. We found that ferroptotic airway epithelial cells exhibit increased tissue plasminogen activator (tPA) secretion, which induces fibroproliferation and excessive accumulation of extracellular matrix proteins in the distal lung parenchyma in a paracrine manner, eventually leading to the pathological remodeling of lung architecture and obliteration of lung tissue.^[^ ^17^ ^]^ Pharmacological inhibition of ferroptosis using ferrostatin‐1 (Ferr‐1) or deferoxamine (DFO) effectively mitigated airway epithelial injury, thereby improving crystalline silica‐induced PF.^[^ ^18^ ^]^ These findings highlight ferroptosis as a promising therapeutic target. However, its precise regulatory mechanisms remain incompletely understood.

Mitochondria maintain iron homeostasis under physiological conditions.^[^ ^19^, ^20^ ^]^ However, mitochondrial dysfunction can lead to the release of free iron into the cytoplasm, triggering the Fenton reaction and generating reactive oxygen species (ROS). ROS can react with polyunsaturated fatty acids in cellular membranes, initiating lipid peroxidation and producing toxic derivatives, such as 4‐hydroxynonenal (4‐HNE) and malondialdehyde (MDA). Mitophagy, a selective form of autophagy, contributes to mitochondrial homeostasis by degrading damaged mitochondria.^[^ ^21^, ^22^ ^]^ PTEN‐induced putative kinase 1 (Pink1) and parkin play crucial roles in maintaining mitochondrial morphology, function, and mitophagy regulation. Pink1 senses mitochondrial injury and recruits Parkin to damaged mitochondria, facilitating its clearance through mitophagy.^[^ ^23^ ^]^ The induction of mitophagy in epithelial cells has been validated to alleviate PF.^[^ ^24^ ^]^ Thus, exploring the potential modulation of mitophagy in the airway epithelium is a promising approach for PF intervention.

Signal transducer and activator of transcription 6 (STAT6) is a multifunctional transcription factor involved in immune regulation and cellular homeostasis.^[^ ^25^ ^]^ It mediates diverse physiological and pathological processes by regulating the transcription of related genes. In our previous studies, we demonstrated that STAT6 regulates fatty acid oxidation in renal epithelial cells, influencing renal fibrosis.^[^ ^26^ ^]^ Additionally, STAT6 maintains cellular homeostasis during acute lung injury (ALI) by indirectly mediating the transcriptional expression of SLC7A11, a crucial subunit of system Xc^−^, in a P53‐dependent manner.^[^ ^18^ ^]^ Although the immunoregulatory functions of STAT6 are well‐characterized, its role in intrinsic cell types, such as airway epithelial cells, remains poorly understood.

In the present study, we investigated the role of STAT6 in maintaining airway epithelial homeostasis during PF progression. We identified STAT6 as a key regulator linking mitophagy and ferroptosis in airway epithelial cells. Specifically, we elucidated a novel mechanism by which STAT6 suppresses Parkin‐mediated mitophagy, leading to mitochondrial dysfunction and enhanced ferroptosis, which exacerbated PF. These findings reveal a critical regulatory axis in PF pathogenesis and suggest new therapeutic opportunities for targeting STAT6 to mitigate PF.

## 2 Results

### 2.1 Mitophagy is Significantly Enhanced in Ferroptotic Airway Epithelial Cells During PF Along With STAT6 Activation

**Figure 1:**
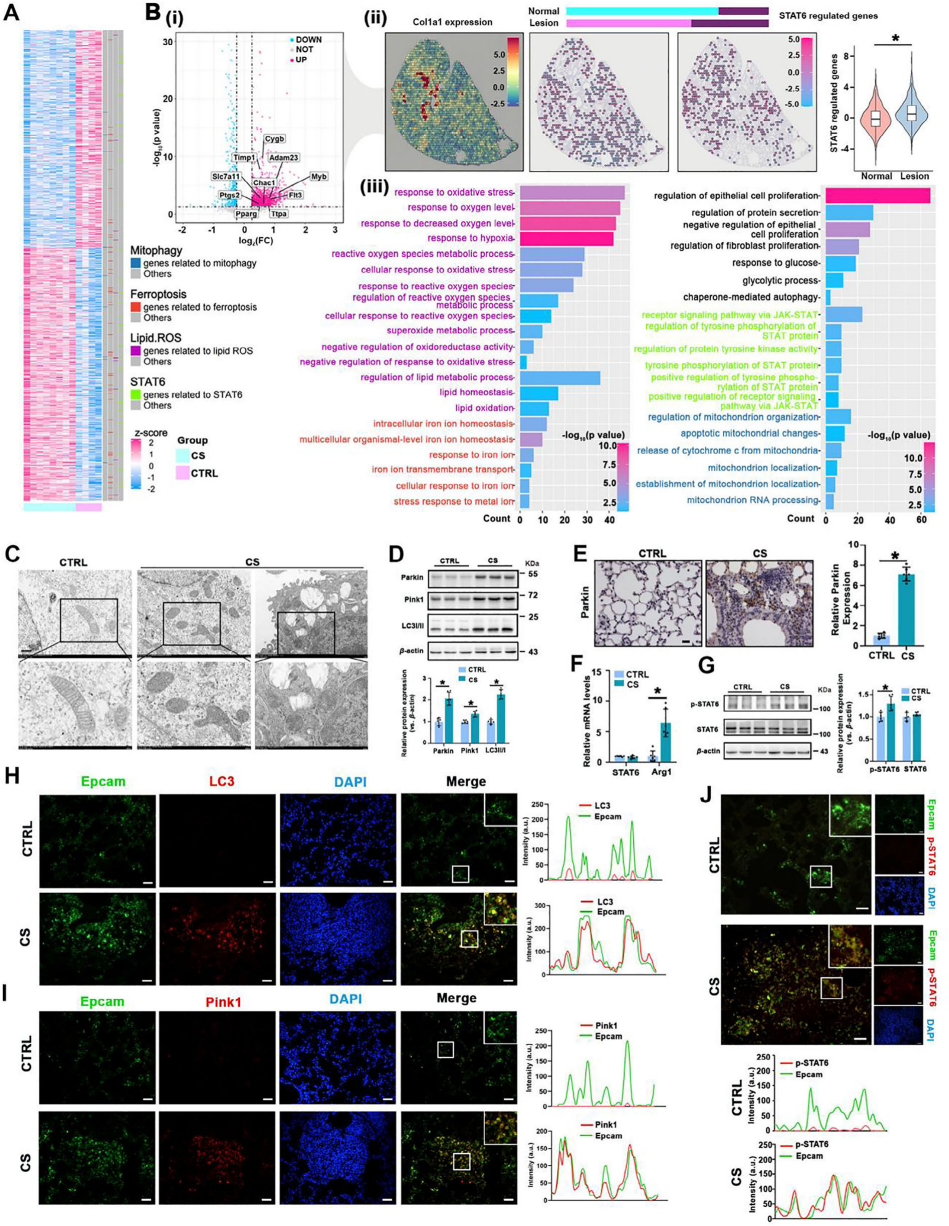
STAT6 activation and mitophagy are significantly enhanced in airway epithelial cells during pulmonary fibrosis. A) Heatmap of differentially expressed genes in the CS‐exposed murine model. Genes associated with specific biological processes were marked. B) Analysis of spatial transcriptome data from a murine model of CS‐induced pulmonary fibrosis. i) The volcano map showed the differential genes between the epithelial cells in the lesion area and the normal area, and ferroptosis‐related genes were labeled. ii) The lesion area was distinguished by the expression level of Col1a1 and expression of STAT6‐related genes in epithelial cells in the lesion and normal areas were labeled. iii) Pathway enrichment of differentially expressed genes in epithelial cells. C) Transmission electron micrographs of the lung tissues focusing on mitochondria, scale bar: 1 µm (upper panel), 500 nm (lower panel). D) The protein expression and quantification of Parkin, Pink1, and LC3I/II in lung tissues. E) IHC staining of Parkin in lung tissue sections was performed and quantified, scale bar: 20 µm. F) Relative mRNA levels of STAT6 and Arg1 in lung tissues. (G) The protein expression and quantification of p‐STAT6 and STAT6 in lung tissues. Representative IF co‐staining and indicated quantification of Epcam & LC3 H) and Epcam & Pink1 I) and J) Epcam & p‐STAT6 in lung tissue sections, profile intensity showing their fluorescence signals, scale bar: 50 µm. The data were presented as means ± SD. *n* = 6 mice per group for C‐J. Statistical analysis was performed using *t*‐test for D‐G. ^*^ *p* < 0.05.

Previously, we demonstrated that ferroptotic airway epithelium augments local fibroblast activation in a paracrine manner, highlighting the critical role of airway epithelium ferroptosis in PF. In this study, we aimed to target epithelial ferroptosis as a therapeutic intervention for PF and uncover the underlying regulatory mechanisms. We first analyzed a CS‐induced PF dataset (GSE32147) and identified DEGs, which revealed significant alterations in genes associated with lipid peroxidation, ferroptosis, mitophagy, and STAT6 regulation (**Figure** **1**A; Figure S1A, Supporting Information). Further investigation of STAT6‐regulated genes in lung tissue RNA‐seq datasets from WT and STAT6 KO mice (GSE1438) revealed a strong positive correlation between STAT6 signaling and PF progression, as shown by GSVA analysis (Figure S1B,C, Supporting Information). To validate these findings, we conducted a spatial transcriptome analysis (STA) using SRP336178. Consistent with the bulk RNA analysis, STA revealed significant upregulation of ferroptosis‐related and STAT6‐regulated genes in epithelial cells within fibrotic lesions, where mitophagy‐ and STAT‐related pathways were also notably enriched (Figure 1Bi–iii).

To further verify the results of our bioinformatics analysis, we established and confirmed a CS‐induced murine PF model characterized by destroyed alveolar structure and excessive collagen deposition (Figure S1D,E, Supporting Information). Evidence of ferroptosis was observed, including elevated 4‐HNE levels, increased iron content, reduced GSH, and upregulated ferroptosis‐related proteins (Figure S1E–G, Supporting Information). IF co‐staining of Epcam and PTGS‐2 in lung sections further confirmed that ferroptosis occurred in the epithelium of fibrotic lesions, which is consistent with our previous findings (Figure S1H, Supporting Information). Transmission electron microscopy of lung tissue from fibrotic lesions revealed impaired mitochondrial morphology, characterized by reduced mitochondrial volume and loss of cristae. Moreover, we detected mitolysosomes, the fusion of mitophagosomes and lysosomes, indicating active mitophagy (Figure 1C). Consistently, key mitophagy‐related proteins, including Parkin, Pink1, and LC3, were significantly upregulated in the CS group (Figure 1D,E). STAT6 activation was also verified by the increased Arg‐1 expression and upregulated p‐STAT6 levels (Figure 1F,G). Additionally, IF co‐staining of Epcam with LC3, Pink1, and p‐STAT6 in fibrotic lesions further confirmed that mitophagy and STAT6 activation occurred in airway epithelial cells (Figure 1H–J). Collectively, these experimental results align with the aforementioned bioinformatics results, demonstrating that mitophagy is significantly enhanced in ferrototic airway epithelial cells during PF, coinciding with STAT6 activation.

### 2.2 Increased Mitophagy and STAT6 Activation are Validated In vitro

**Figure 2:**
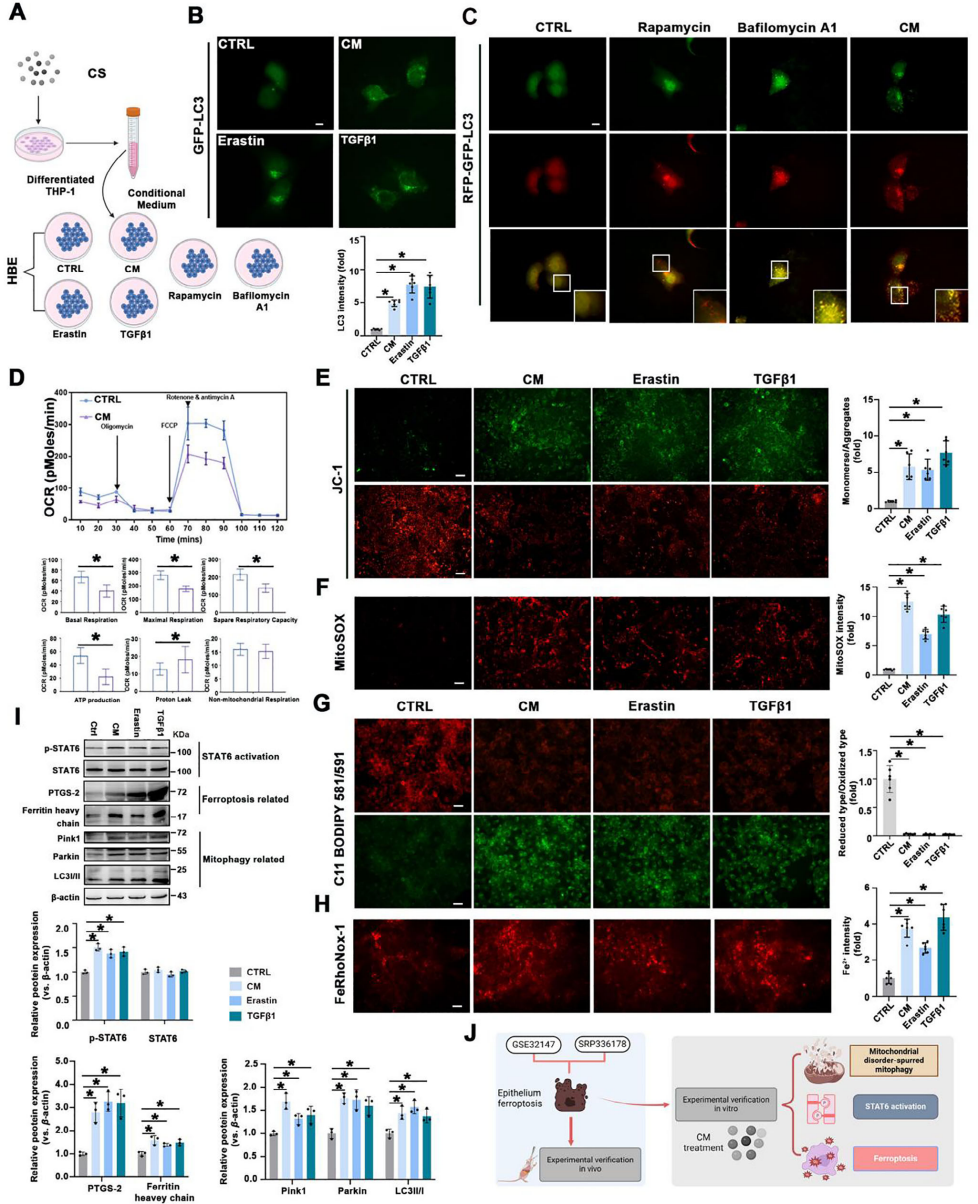
Mitochondrial disorder‐spurred mitophagy is involved in CS‐induced ferroptosis along with STAT6 activation. A) Schematic diagram of cell experiment. B) Representative images of IF staining of GFP‐LC3 in HBE cells with indicated treatments and quantification below, scale bar: 5 µm. Erastin (10 µm), TGFβ1 (5 ng mL^−1^). C) HBE cells were transfected with a tandem RFP‐GFP‐LC3 construct for 24 h and then either left untreated or separately treated with Bafilomycin A1 (100nm, 4 h), Rapamycin (1 µm, 24 h), and CM. Representative image of autophagosomes (yellow puncta on overlay) and autolysosomes (RFP puncta on overlay) in HBE cells, scale bar: 5 µm. D) Oxygen consumption rate (OCR) in HBE cells with the indicated treatment. E) The mitochondrial membrane potential in HBE cells with different treatments was evaluated and quantified, scale bar: 50 µm. F) Mitochondrial superoxide in HBE cells with different treatments was evaluated and quantified, scale bar: 20 µm. G) Lipid ROS production and H) intracellular Fe^2+^ levels in HBE cells were evaluated and quantified, scale bar: 50 µm. I) The indicated protein expression was detected by immunoblot analysis and quantified. J) The flowchart and schematic diagram of this figure. The data were presented as means ± SD. *n* = 6 for (B, C), and (E–H), *n* = 3 for D and I. Statistical analysis was performed using one‐way ANOVA with Tukey's post hoc test for B and E‐I, *t*‐test for D. ^*^ *p* < 0.05.

Building on the aforementioned results from the bioinformatics analyses and in vivo experiments, we next established an in vitro model to further determine these phenomena in airway epithelial cells. Differentiated THP1 cells were treated with CS, and the resulting conditional medium (CM) was collected and applied to HBE cells. A schematic representation of the treatment protocol is shown in **Figure** **2**A. The result showed that LC3 expression was significantly increased following CM treatment, consistent with the effect of Erastin and TGFβ1, which served as positive controls (Figure 2B). An RFP‐GFP‐LC3 tandem fluorescence assay was used to assess autophagy in HBE cells exposed to CM. The GFP signal, which is typically quenched upon autophagosome‐lysosome fusion, and the RFP signal, which remains stable, allowed for the visualization of autophagosomes (yellow puncta) and autolysosomes (red puncta). CM treatment resulted in a significant increase in the red puncta, indicating enhanced autophagic flux and autolysosome formation, similar to the effects of rapamycin, a classical autophagy inducer (Figure 2C). Mitochondrial damage was also detected. Seahorse analysis showed suppressed mitochondrial respiration (OCR) after CM treatment (Figure 2D). JC‐1 and MitoSOX staining indicated impaired mitochondrial membrane potential and increased mitochondrial ROS levels (Figure 2E,F). Ferroptosis was assessed using C11 BODIPY 581/591 and FeRhoNox‐1 staining. As expect, CM significantly forced ferroptosis as demonstrated by increased lipid peroxidation and Fe^2+^ accumulation, consistent with the effect of Erastin and TGFβ1 (Figure 2G,H). Immunoblot analysis further confirmed the concurrent upregulation of ferroptosis markers, increased levels of mitophagy‐related proteins (Parkin, Pink1, and LC3), and STAT6 activation in HBE cells exposed to CM (Figure 2I). Together, these in vitro findings suggest that CM stimulation enhances ferroptosis and mitophagy in epithelial cells, accompanied by STAT6 activation (Figure 2J), which is consistent with our in vivo results.

### 2.3 STAT6 Deficiency Alleviates PF with Enhanced Mitophagy and Suppressed Epithelial Ferroptosis

**Figure 3:**
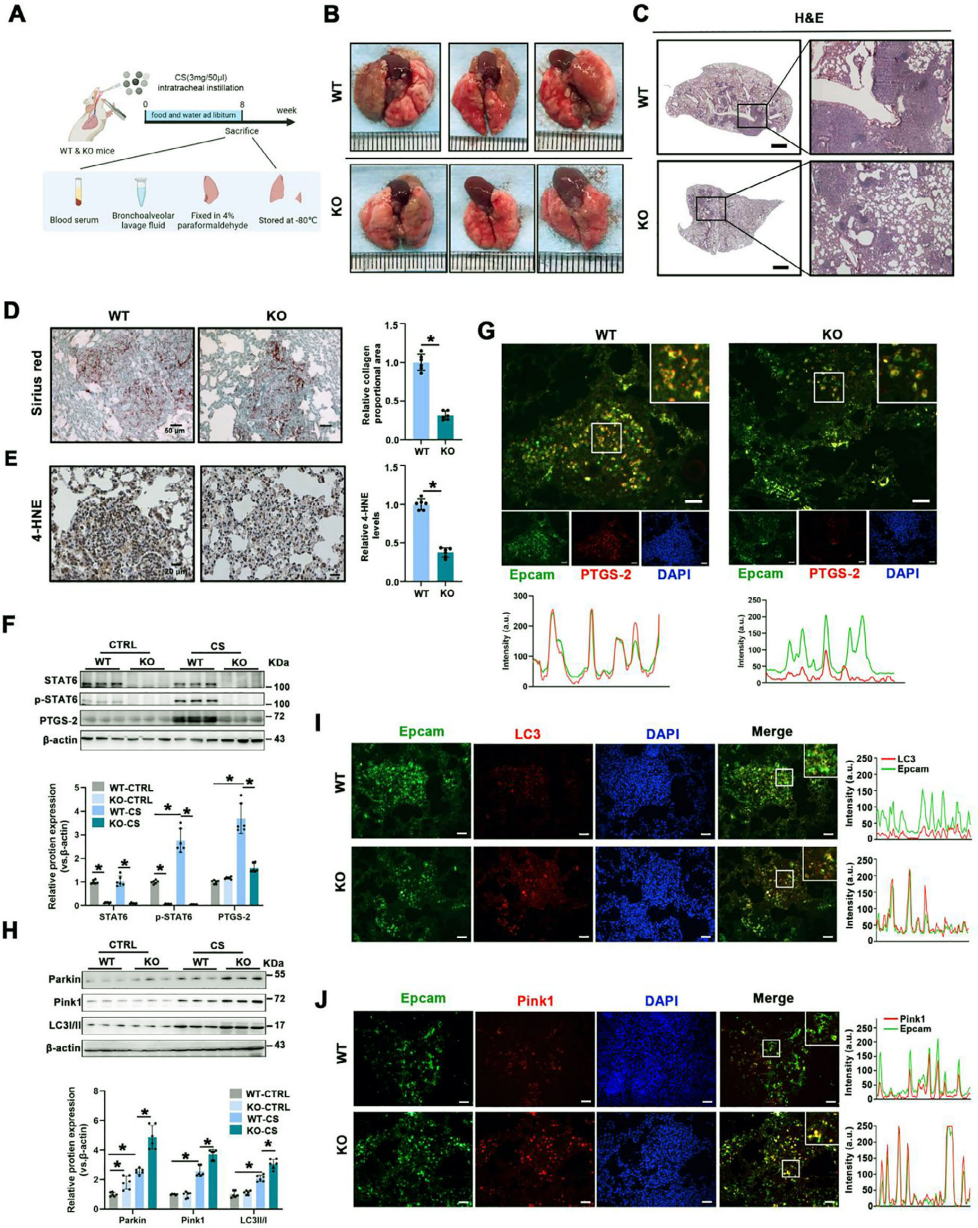
STAT6 deficiency promotes airway epithelium mitophagy and suppresses ferroptosis in CS‐induced lung injury. A) Schematic diagram of the in vivo experiment. B) Representative images of whole lung tissue from the indicated group. C) Representative H&E‐stained lung sections from WT and STAT6 KO mice, scale bar: 1 mm. D) Representative images of Sirius Red staining of lung tissue sections, and relative collagen quantification were shown in the right panel, scale bar 50 µm. E) IHC staining of 4‐HNE in lung tissue sections was performed and quantified, scale bar: 20 µm. F) The protein expression and quantification of STAT6, p‐STAT6, and PTGS‐2 in lung tissue. G) Representative IF co‐staining of Epcam & PTGS‐2 in lung tissue sections, scale bar: 50 µm. H) The protein expression related to mitophagy (Parkin, Pink1, LC3) was measured by immunoblot analysis and quantified. Representative IF co‐staining of Epcam & LC3 I) and Epcam & Pink1 J) in lung tissue sections, profile intensity showing their fluorescence signals, scale bar: 50 µm. The data were presented as means ± SD. *n* = 6 mice per group for (B–J). Statistical analysis was performed using *t*‐test for (D and E), two‐way ANOVA with Tukey's post hoc test for F, H. ^*^ *p* < 0.05.

Since STAT6 activation is likely to promote PF, we employed STAT6 KO mice in a PF model to further investigate its role in mitophagy and ferroptosis (**Figure** **3**A). Compared to WT mice, STAT6 deficiency significantly alleviated CS‐induced PF, manifesting as fewer pulmonary nodules, reduced alveolar structure impairment, and decreased collagen deposition (Figure 3B–D; Figure S2A,B, Supporting Information). As expected, ferroptosis was mitigated in CS‐exposed STAT6 KO mice, as assessed by the lower levels of 4‐HNE and PTGS‐2 (Figure 3E,F; Figure S2C, Supporting Information). IF co‐staining of PTGS‐2 and Epcam consistently showed suppressed epithelial ferroptosis in STAT6 KO mice (Figure 3G; Figure S2D, Supporting Information). Next, we assessed mitophagy. STAT6 KO mice exhibited increased levels of Parkin, Pink1, and LC3 after CS inhalation, suggesting enhanced mitophagy, which was further confirmed by co‐staining of LC3 & Epcam and Pink1 & Epcam (Figure 3H–J; Figure S2E,F, Supporting Information). These data suggest that STAT6 is a crucial regulator of ferroptosis and mitophagy in PF.

Given the complexity of the airway epithelium and the challenges in generating conditional knockout mice, lentiviral vectors were used to manipulate STAT6 expression specifically in the airway epithelium via intratracheal instillation. Lentiviral‐mediated knockdown of STAT6 in WT mice was achieved using shRNA constructs, whereas STAT6 overexpression in KO mice was induced using lentiviral vectors carrying STAT6 (Figure S3A,G, Supporting Information). The results showed that compared to the indicated control mice, epithelial STAT6 depletion significantly alleviated CS‐induced PF, with inhibited airway epithelium ferroptosis and enhanced mitophagy (Figure S3B–F, Supporting Information). Conversely, STAT6 overexpression in the epithelium exacerbated mitophagy inhibition, increased ferroptosis, and worsened PF (Figure S3H–L, Supporting Information). These data suggest that STAT6 contributes to PF by inhibiting mitophagy and promoting ferroptosis.

### 2.4 STAT6 Negatively Regulates Mitophagy to Aggravate Ferroptosis In Vitro

**Figure 4:**
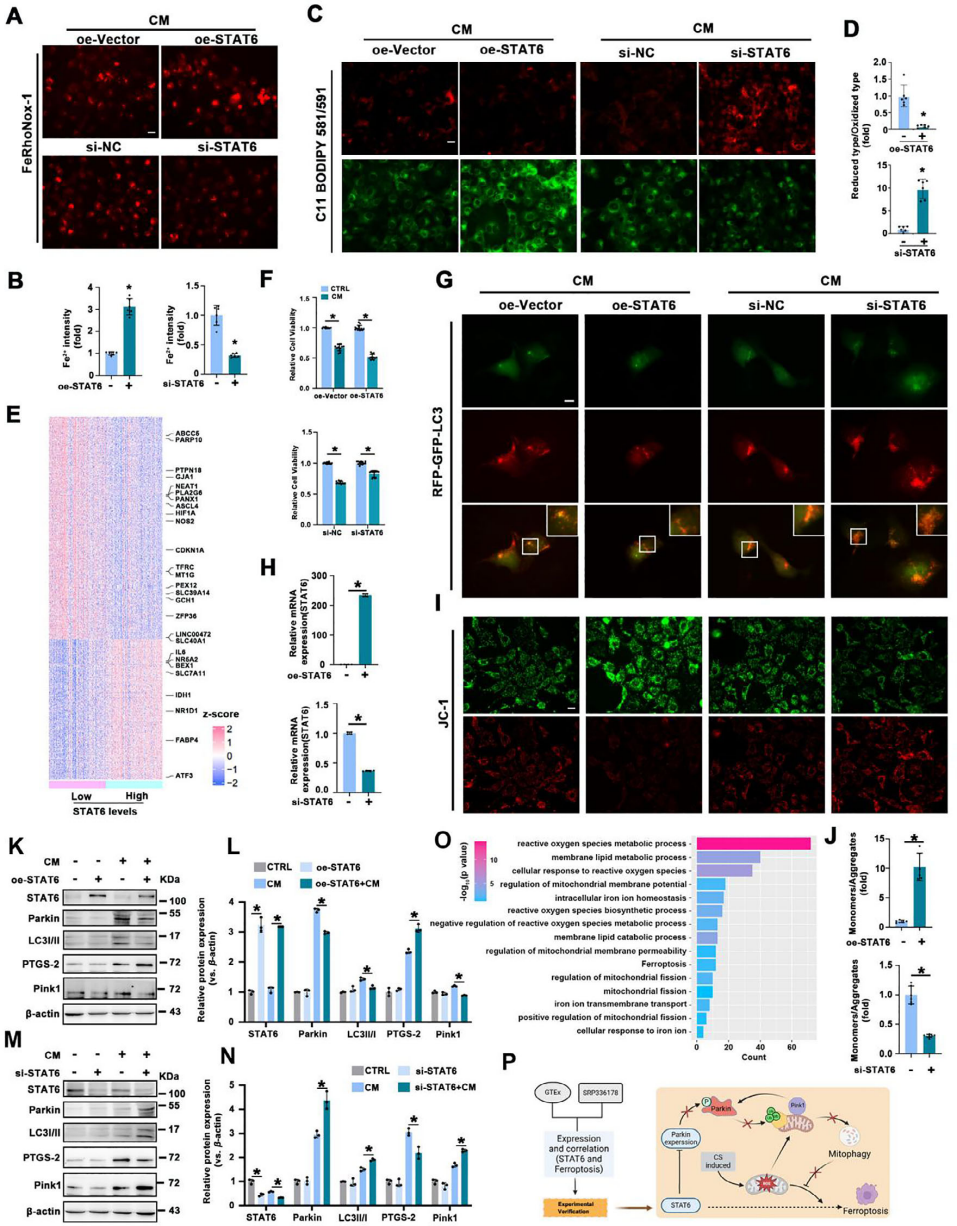
STAT6 mediates ferroptosis through negative regulation of mitophagy. A–B) Intracellular Fe^2+^ levels and quantification, scale bar: 20 µm. C,D) Lipid ROS production in HBE cells was evaluated and quantified, scale bar: 20 µm. E) Differentially expressed genes (DEG) between STAT6 high‐low expression groups in GTEx samples, with genes associated with ferroptosis annotated. F) Relative cell viability of HBE cells was measured by MTT assay. G) HBE cells were transfected with a tandem mRFP‐GFP‐LC3 construct for 24 h and with the indicated treatment. Representative image of autophagosomes (yellow puncta on overlay) and autolysosomes (RFP puncta on overlay), scale bar: 5 µm. H) Relative mRNA levels of STAT6 in the HBE cells with the indicated treatment. I,J) Mitochondrial membrane potential of HBE cells was detected by JC‐1 staining and quantified, scale bar: 20 µm. K–N) The protein expression and quantification of STAT6, Parkin, LC3, PTGS‐2, and Pink1 in HBE cells with indicated treatments. O) Enrichment of pathways with high and low expression of STAT6 in epithelial cells. P) A flowchart describing how STAT6 promotes ferroptosis through bioinformatics analysis and conducting experimental verification. The results were presented as means ± SD. *n* = 6 for (A–D, G, I, J), *n* = 4 for H, *n* = 3 for (K–N), *n* = 12 for (F). Statistical analysis was performed using *t*‐test for (B, D, H, and J), two‐way ANOVA with Tukey's post hoc test for (F, L and N). ^*^ *p* < 0.05.

These results establish the crucial role of STAT6 in PF. To further investigate its regulatory function in epithelial cells, HBE cells were transfected with a STAT6 plasmid or siRNA to upregulate or downregulate STAT6 expression, respectively. Following STAT6 regulation, the HBE cells were treated with CM. The results showed that overexpression of STAT6 enhanced CM‐induced Fe^2+^ accumulation and lipid peroxidation, whereas STAT6 knockdown attenuated these effects (**Figure** **4**A–D; Figure S4A,B, Supporting Information). GTEx analysis consistently revealed a correlation between STAT6 activation levels and the expression of ferroptosis‐related genes, reinforcing the link between STAT6 and ferroptosis (Figure 4E). MTT analysis further indicated that STAT6 overexpression forced CM‐eroded cell viability, which was reversed by STAT6 knockdown (Figure 4F). Next, mitophagy classical biomarkers and mitochondrial dysfunction were evaluated. The RFP‐GFP‐LC3 tandem fluorescence assay showed that STAT6 overexpression significantly reduced the number of red puncta, whereas STAT6 knockdown increased their number, indicating that STAT6 negatively regulates mitophagy (Figure 4G; Figure S4C, Supporting Information). JC‐1 staining, used to detect mitochondrial membrane potential, was conducted to evaluate mitochondrial function. CM‐induced mitochondrial dysfunction was exacerbated by STAT6 overexpression and alleviated by STAT6 knockdown (Figure 4I,J; Figure S4D, Supporting Information). These findings are consistent with the in vivo results shown in Figure 3, which showed that mitophagy was increased in STAT6 KO mice with a benefit to mitochondrial function. The efficiency of STAT6 manipulation was confirmed using qPCR and western blotting (Figure 4H,K,M). As expected, results from immunoblot analysis further corroborated that STAT6 negatively regulated mitophagy and promoted ferroptosis, as evidenced by decreased levels of Parkin, Pink1, and LC3 levels, while increased PTGS‐2 in the STAT6 overexpression group, which were reversed in the STAT6 knockdown group (Figure 4K–N). GSEA of STA also supported the negative regulation of STAT6 in mitophagy (Figure 4O).

Mitophagy plays a critical role in the elimination of damaged mitochondria, thereby preventing ROS accumulation and mitigating ferroptosis. To further investigate the relationship between mitophagy and ferroptosis, ferroptosis inducers and inhibitors were used. The results showed that the ferroptosis inhibitors Ferr‐1 and DFO significantly alleviated CS‐induced PF without affecting STAT6 signaling or mitophagy (Figures S5 and S6, Supporting Information). Similarly, in vitro experiments showed that the ferroptosis inducer Erastin exacerbated CM‐induced ferroptosis in HBE cells, whereas Ferr‐1 and DFO effectively reversed ferroptosis, with no significant impact on STAT6 and mitophagy (Figures S7 and S8, Supporting Information). Taken together, these data suggest that STAT6 activation negatively regulates mitophagy. Whether mitophagy is upstream of ferroptosis and how to regulate ferroptosis is our next step (Figure 4P).

### 2.5 STAT6 Directly Binds to the PRKN Promoter to Suppress Mitophagy and Enhance Ferroptosis

**Figure 5:**
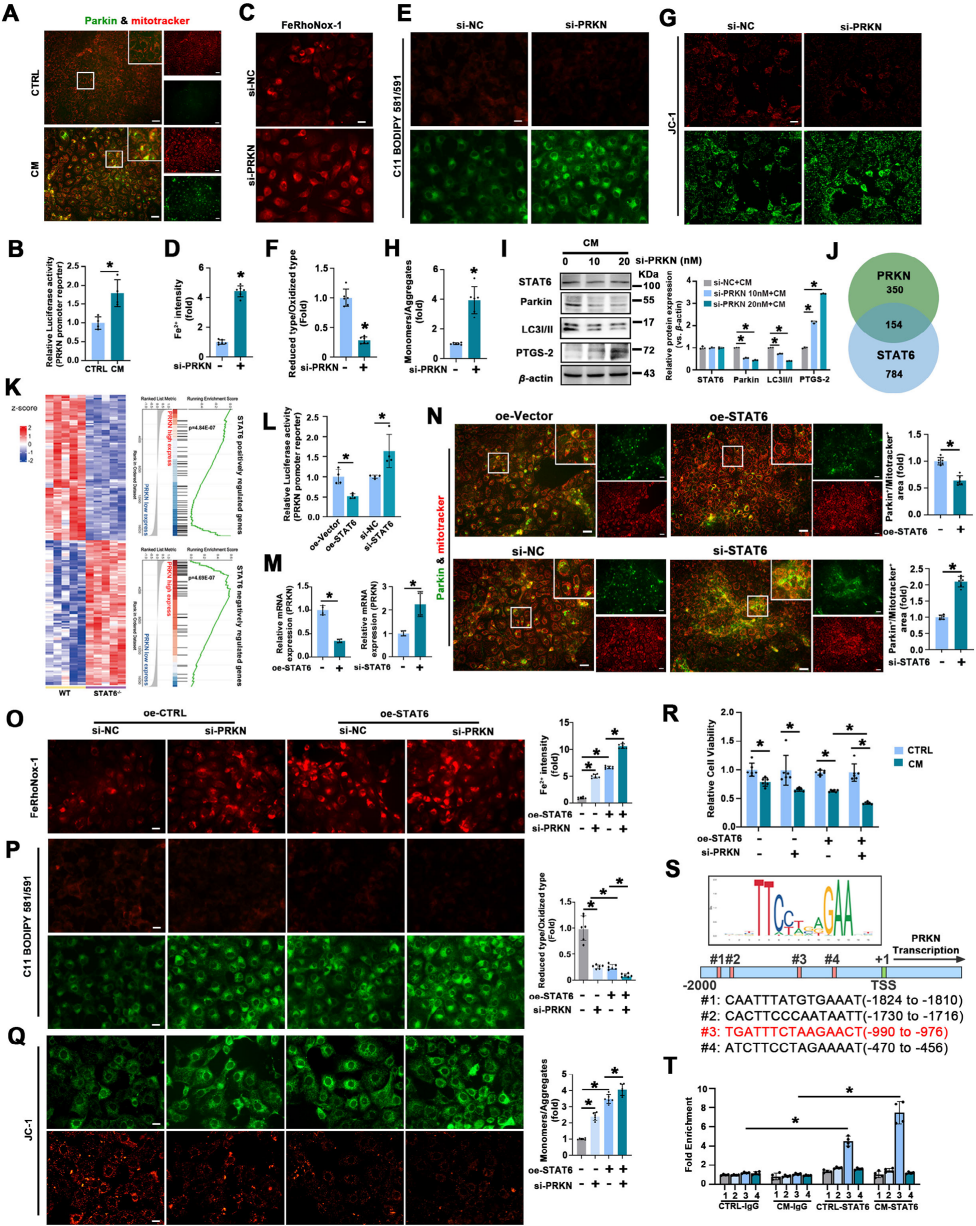
STAT6 negatively regulates mitophagy in a PRKN dependent manner. A) Representative IF co‐staining of Parkin & mitotracker in HBE cells, scale bar: 50 µm. B) Human PRKN promoter was cloned into pGL3.0‐luc vector. HBE cells were either transfected with empty vector or the construct along with Renilla luciferase reporter for 24 h, and followed by another 72 h CM treatment, relative luciferase activity was evaluated. C,D) Intracellular Fe^2+^ levels in HBE cells were evaluated and quantified, scale bar: 20 µm. E,F) Lipid ROS production in HBE cells was evaluated and quantified, scale bar: 20 µm. G,H) Mitochondrial membrane potential was evaluated and quantified, scale bar: 20 µm. I) The protein expression and quantification of Parkin, LC3, and PTGS‐2 in HBE cells with indicated treatments. J) The Venn diagram showed the overlap 154 genes between DEGs grouped by PRKN and STAT6. K) Left panel, heat map showed genes negatively or positively regulated by STAT6; Right panel, GSEA plot showed enrichment of “STAT6 positively regulated genes” in PRKN low expression group and “STAT6 negatively regulated genes” in PRKN high expression group. L) HBE cells with STAT6 overexpression or knockdown were transfected with PRKN promoter luciferase reporter along with Renilla luciferase reporter for 24 h followed by exposure to CM for 72 h, then the luciferase assay was employed to evaluate the relative luciferase activity. M) Relative mRNA level of PRKN in HBE cells with indicated treatments. N) Representative IF co‐staining of Parkin & mitotracker in HBE cells with indicated treatment, and the bar graph represents the quantification data of yellow density (co‐localization), scale bar: 50 µm. O) Intracellular Fe^2+^ levels and P) Lipid ROS production and Q) mitochondrial membrane potential in HBE cells with indicated treatments were evaluated and quantified, scale bar: 20 µm. R) Relative cell viability was measured by MTT assay. S,T) Identification of four binding sites in the promoter of PRKN. The potential site of STAT6 binding to PRKN promoter was detected by ChIP assay in HBE cells with or without CM treatment. The results were presented as means ± SD. *n* = 6 for (A), (C–H) and (N–R), *n* = 4 for (B, L, M and T), *n* = 3 for (I). Statistical analysis was performed using *t*‐test for (B, D, F, H, L, M and N), one‐way ANOVA or two‐way ANOVA with Tukey's post hoc test for (I), (O–R) and (T). ^*^ *p* < 0.05.

Next, we explored the role of STAT6 in mitophagy and the detailed regulatory mechanisms. Parkin (encoded by PRKN) is a critical regulator of mitophagy, and its upregulation has been associated with PF, as shown in the above results. Consistently, IF co‐staining of Parkin and mitotracker showed increased co‐localization of Parkin and mitotracker following CM treatment, as evidenced by yellow fluorescence in the merged channel (**Figure** **5**A). Additionally, dual‐luciferase reporter assays revealed that CM administration significantly increased PRKN promoter activity (Figure 5B). To explore the functional role of Parkin, we interfered its expression in HBE cells, and the interference efficiency was validated by immunoblot analysis (Figure 5I). As expected, Parkin knockdown significantly augmented CM‐induced Fe^2+^ accumulation and lipid peroxidation (Figure 5C–F; Figure S4E,F, Supporting Information). Consistently, JC‐1 staining demonstrated that Parkin interference intensified CM‐induced mitochondrial dysfunction (Figure 5G,H; Figure S4G, Supporting Information). Differential analysis of GTEx lung tissue data was performed to stratify the median STAT6 and PRKN expression levels. The analysis identified 154 overlapping DEGs between the STAT6 and PRKN groups (Figure 5J). To further clarify the relationship between STAT6 and PRKN, gene sets from STAT6 KO mice were used to apply GSEA to the GTEx samples grouped by median PRKN expression. Heatmap visualization of the top 200 DEGs (100 upregulated and 100 downregulated genes) from GSE1438 revealed distinct gene expression patterns (Figure 5K, left). GSEA further revealed that the genes positively regulated by STAT6 were significantly enriched in the low PRKN expression group, whereas the genes negatively regulated by STAT6 were enriched in the high PRKN expression group (Figure 5K, right). The results of qRT‐PCR and luciferase reporter assay confirmed an inverse correlation between STAT6 and PRKN (Figure 5L,M). IF co‐staining of Parkin and mitotracker also showed that STAT6 overexpression suppressed CM‐induced Parkin expression, whereas STAT6 knockdown enhanced it (Figure 5N). These findings suggest that STAT6 inhibits Parkin‐mediated mitophagy, which in turn enhances ferroptosis.

To assess the functional consequences of Parkin suppression, we investigated the effects of Parkin depletion on ferroptosis and mitochondrial dysfunction. Parkin knockdown exacerbated CM‐induced mitochondrial dysfunction and ferroptosis, as evidenced by increased iron accumulation, lipid peroxidation, and reduced cell viability, which were further amplified by STAT6 overexpression (Figure 5O–R). The detailed regulation of STAT6 in Parkin expression was further identified, and four candidate binding sites for STAT6 binding were found in the PRKN promoter (Table S3, Supporting Information). The potential site of STAT6 binding to PRKN promoter was detected using a ChIP assay. The results showed that there was direct binding between STAT6 and PRKN, predominantly at the third binding site (−990 to −976 bp; Figure 5S,T). These data show that STAT6 regulates ferroptosis by directly binding to the PRKN promoter to inhibit mitophagy.

### 2.6 Suppression of PRKN‐Mediated Mitophagy Exacerbates CS‐Induced Epithelium Ferroptosis and Fibrosis

**Figure 6:**
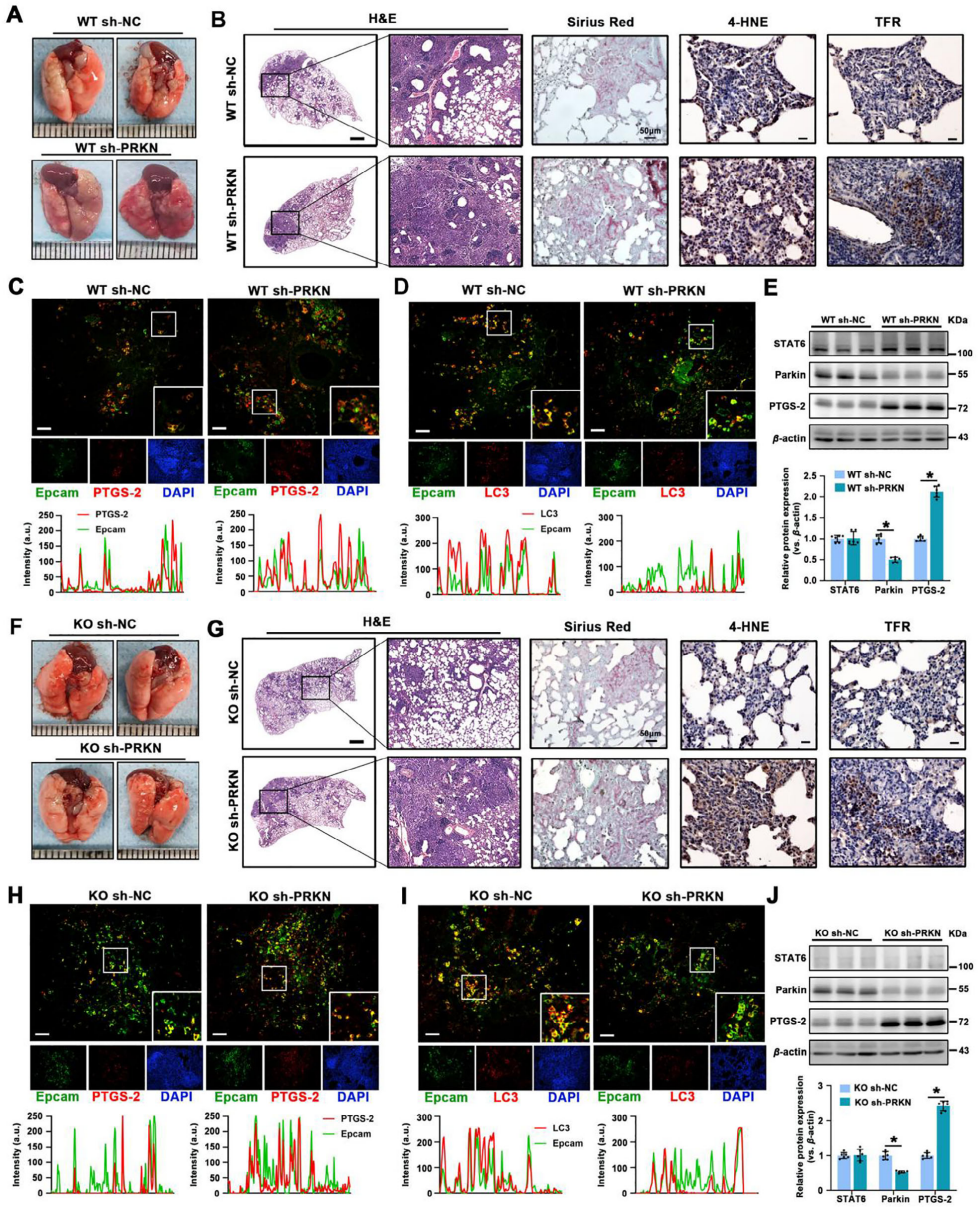
Parkin, as a downstream gene with negative regulation of STAT6, plays a protective role in ferroptosis. A,F) Representative images of whole lung tissue from the indicated group. B,G) Representative images of H&E (scale bar: 1mm), Sirius Red staining (scale bar: 50 µm), and IHC staining (scale bar: 20 µm) of 4‐HNE and TFR in lung tissue sections. C,H) Representative IF co‐staining of Epcam & PTGS‐2 and D,I) Epcam & LC3 in lung tissue sections, profile intensity showing their fluorescence signals, scale bar: 50 µm. E,J) The protein expression and quantification of STAT6, Parkin, and PTGS‐2 in lung tissue. The results were presented as means ± SD. *n* = 6 mice per group for A‐J. Statistical analysis was performed using *t*‐test for E and J. ^*^ *p* < 0.05.

To further elucidate the role of Parkin in PF, we used a CS‐induced murine model, with both WT and STAT6 KO mice intratracheally instilled lentivirus‐sh‐PRKN to specifically suppress PRKN expression in the airway epithelium. The efficiency of PRKN depletion was verified by western blot analysis (**Figure** **6**E,J). Results showed that mice with PRKN deficiency manifested severe PF in both WT and STAT6 KO mice (Figure 6A,F). H&E and Sirius Red staining consistently showed more severe alveolar structure impairment and increased collagen fiber deposition. IHC staining for 4‐HNE and TFR showed that PRKN deficiency significantly aggravated CS‐induced ferroptosis (Figure 6B,G,E,J; Figure S4I–N, Supporting Information). As expected, IF co‐staining of PTGS‐2 and Epcam suggested that epithelial ferroptosis was markedly enhanced in PRKN‐deficient mice exposed to CS (Figure 6C,H). Furthermore, co‐staining of Epcam and LC3 revealed that CM‐induced LC3 expression was notably inhibited by PRKN depletion (Figure 6D,I). Taken together, these data suggest that suppression of PRKN‐mediated mitophagy exacerbated epithelial ferroptosis and contributed to the progression of PF in both WT and STAT6 KO mice.

### 2.7 Rifabutin is the Novel Candidate as a Potential STAT6 Inhibitor to Promote Mitophagy and Suppress Ferroptosis

**Figure 7:**
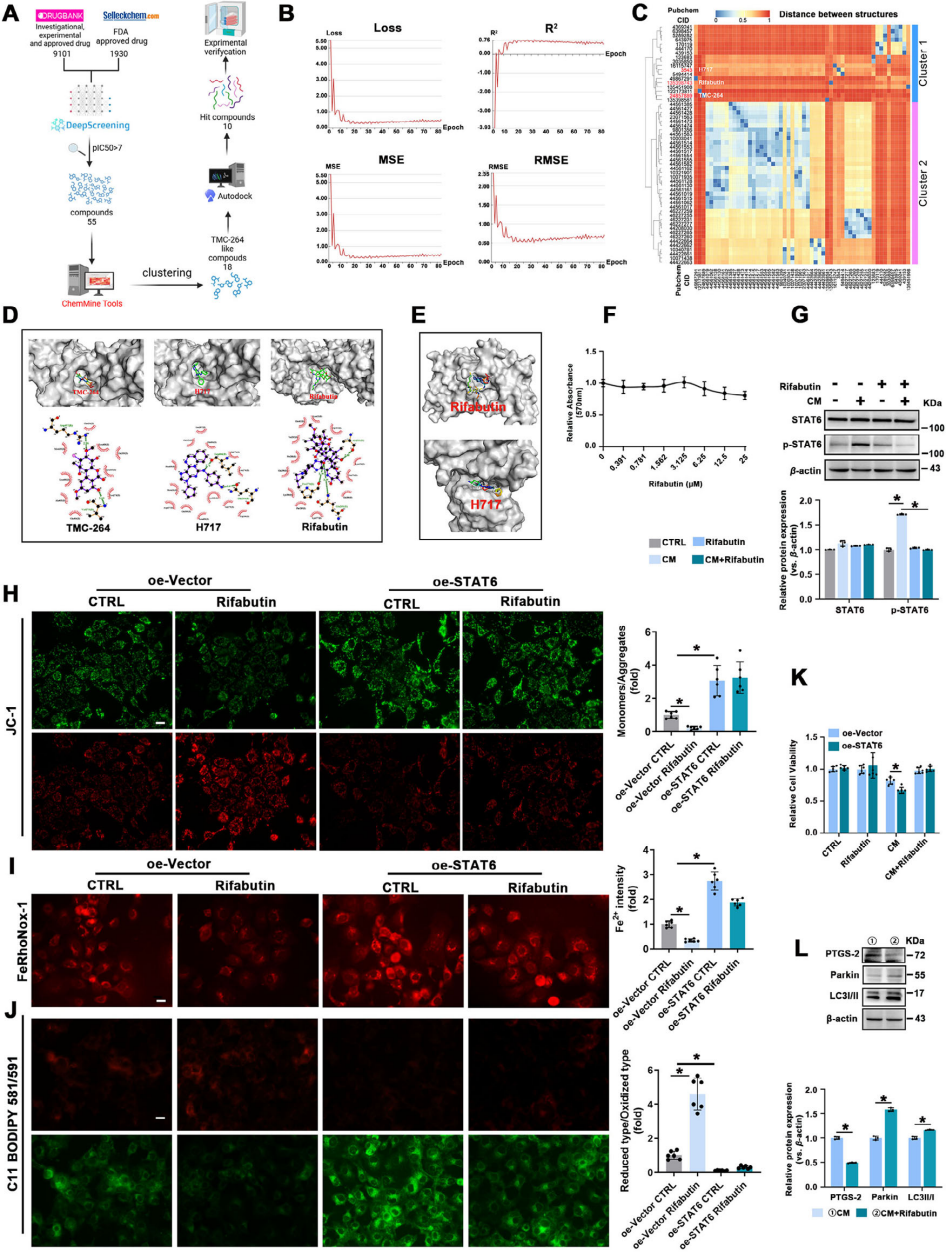
Rifabutin is identified as a potential STAT6 inhibitor to promote mitophagy. A) Flowchart of virtual screening. B) Statistical parameters of the deep learning screening model. R^2^, R‐squared; MSE, mean squared error; RMSE, root MSE. C) Heatmap and clustering tree revealing the clustering results of screened compounds and TMC‐264 based on atom pair descriptors. D) 3D and 2D images of virtual docking between TMC‐264/H717/Rifabutin and STAT6. The green lines in the 2D images represent hydrogen bonds. E) The 3D structure of the superimposition of the docked ligand with its original X‐ray crystal structure. F) Relative cell viability was measured by MTT assay. G) The protein expression of STAT6 and p‐STAT6 in HBE cells with the indicated treatment. H) The mitochondrial membrane potential of HBE cells was detected by JC‐1 staining, scale bar: 20 µm. I) Representative images of FeRhoNox‐1 and J) C11 BODIPY 581/591 staining, scale bar: 20 µm. K) Relative cell viability of HBE cells with the indicated treatment. L) The protein expression of PTGS‐2, Parkin, and LC3 in HBE cells. The results were presented as means ± SD. *n* = 3 for (F, G and L), *n* = 6 for (H–K). Statistical analysis was performed using one‐way or two‐way ANOVA with Tukey's post hoc test for (G–L). ^*^ *p* < 0.05.

Building on our finding that STAT6 inhibition protected against PF, we aimed to identify potential drugs targeting STAT6 using a systematic screening workflow (**Figure** **7**A). A predictive model for STAT6 inhibition was developed using DeepScreening, which demonstrated robust performance with a Loss value of 0.27, R squared (R^2^) of 0.76, mean squared error (MSE) of 5.50, and root MSE (RMSE) of 2.35 (Figure 7B). Using this model, 9101 drugs from DrugBank and 1930 FDA‐approved drugs from Selleckchem were screened. As a reference, AS1517499, a known STAT6 inhibitor with a pIC50 of 7.68, was included in the analysis, leading to the identification of 55 drugs with predicted pIC50 values of >7.^[^ ^27^ ^]^

To refine these candidates, we used TMC‐264, a molecule that reduces STAT6 phosphorylation and blocks the formation of phosphorylated STAT6‐DNA complexes, as a reference for structural similarity.^[^ ^28^ ^]^ Among the 55 shortlisted drugs, 18 compounds were identified as structurally similar to TMC‐264 based on atom‐pair descriptors (Figure 7C). Molecular docking analysis of these compounds revealed 10 candidates with binding energies lower than that of TMC‐264 (Table S4, Supporting Information), including a purine derivative, 2‐[trans‐(4‐aminocyclohexyl)amino]‐6‐(benzyl‐amino)‐9‐cyclopentylpurine (H717), which exhibited the lowest binding energy and the highest predicted pIC50. Rifabutin, another hit compound, demonstrated favorable binding energy compared to other approved drugs (Figure 7D). Re‐docking experiments further validated rifabutin and H717, with RMSD values of 0.00 and 1.18 Å, respectively (Figure 7E). Considering its clinical availability, rifabutin was identified as a promising STAT6 inhibitor for PF therapy.

Next, we validated the in vitro effects of rifabutin using CM‐treated HBE cells. MTT assay confirmed the cytotoxic tolerance of rifabutin at the experimental doses (Figure 7F). Western blot analysis demonstrated that rifabutin effectively inhibited STAT6 activation (Figure 7G). JC‐1 staining revealed that rifabutin alleviated mitochondrial dysfunction, which was abrogated by STAT6 overexpression (Figure 7H). Rifabutin also inhibited ferroptosis as demonstrated by decreased Fe^2+^ accumulation and lipid peroxidation, whereas this effect was eroded by STAT6 overexpression (Figure 7I,J). The cell viability assay further confirmed that rifabutin mitigated ferroptosis by inducing STAT6‐mediated mitophagy (Figure 7K). Immunoblot analysis also showed that rifabutin significantly suppressed CM‐induced PTGS‐2 expression along with the induction of Parkin and LC3 (Figure 7L). In conclusion, rifabutin is a promising STAT6 inhibitor that prevents ferroptosis through the induction of mitophagy.

### 2.8 Rifabutin Ameliorates Ferroptosis and Improves PF in CS Exposed Mice

**Figure 8:**
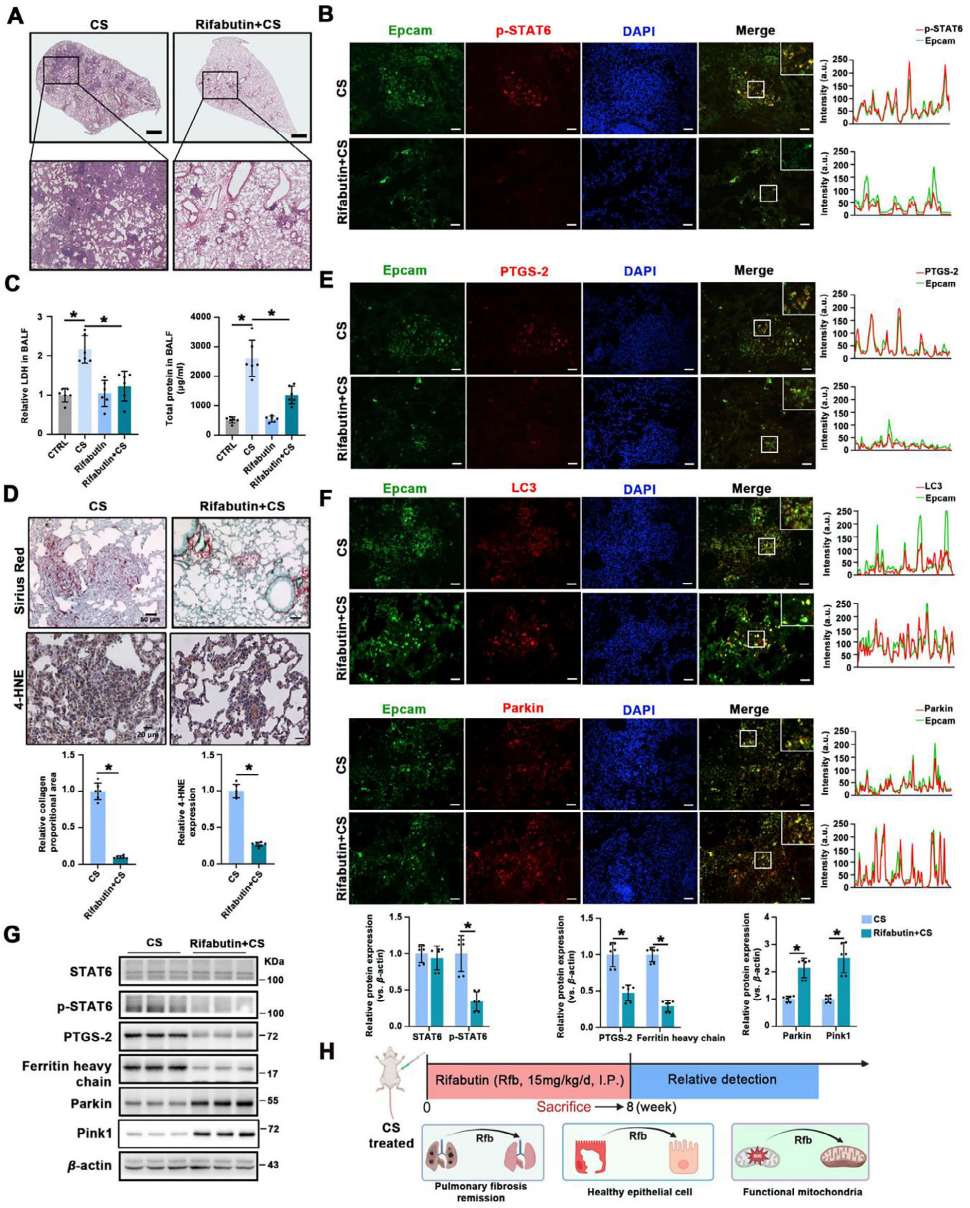
Rifabutin inhibits ferroptosis and alleviates CS‐induced pulmonary fibrosis. A) Representative H&E‐stained lung sections from CS group and Rifabutin+CS group mice, scale bar: 1 mm. B) Representative IF co‐staining of Epcam & p‐STAT6 in lung tissue sections, profile intensity showing their fluorescence signals, scale bar: 50 µm. C) Relative protein and LDH content in BALF of the indicated group. D) Representative images of Sirius Red (scale bar: 50 µm) and IHC (scale bar: 20 µm) staining (4‐HNE) of lung tissue sections and quantification. E) Representative IF co‐staining of Epcam & PTGS‐2, F) Epcam & LC3, and Epcam & Parkin in lung tissue sections, profile intensity showing their fluorescence signals, scale bar: 50 µm. G) The protein expression and quantification of STAT6, p‐STAT6, PTGS‐2, ferritin heavy chain, Parkin, and Pink1 in lung tissue. H) The schematic diagram of Rifabutin's effect on CS‐induced pulmonary fibrosis. The data were presented as means ± SD. *n* = 6 mice per group for (A–G). Statistical analysis was performed using two‐way ANOVA with Tukey's post hoc test for (C), *t*‐test for (D and G). ^*^ *p* < 0.05.

Following the in vitro study, we investigated the effects of rifabutin on CS‐induced mice PF. H&E staining showed that rifabutin significantly alleviated the morphological changes induced by CS (**Figure** **8**A). IF staining and immunoblot analysis suggested inhibition of epithelial p‐STAT6 after rifabutin administration (Figure 8B,G). Rifabutin restored CS‐induced LDH and protein levels in BALF, indicating reduced epithelial injury (Figure 8C). Sirius Red staining further confirmed that rifabutin treatment significantly reduced collagen deposition and fibrotic lesions in the lungs (Figure 8D). Consistently, rifabutin effectively suppressed CS‐induced epithelial ferroptosis, as indicated by the decreased levels of ferroptosis‐related markers such as 4‐HNE, PTGS‐2, and FTH1 (Figure 8D,E,G). IF staining and immunoblot analysis showed that rifabutin upregulated mitophagy, as demonstrated by increased LC3, Parkin, and Pink1 expression (Figure 8F,G). Collectively, these data show that rifabutin inhibits ferroptosis and alleviates CS‐induced PF in vivo by inhibiting STAT6 and inducing mitophagy (Figure 8H).

## 3 Discussion

Over the past decades, the prevalence and incidence of PF have steadily increased, posing significant social and economic burdens.^[^ ^29^ ^]^ As PF is a fatal lung disease with a poor prognosis, there is an urgent need to elucidate its underlying mechanisms to identify effective therapeutic strategies. Silica, a prevalent exogenous toxin, has been implicated in diseases such as pneumoconiosis, PF, and lung cancer.^[^ ^30^ ^]^ In this study, we used a CS‐induced PF model to elucidate the detailed regulatory mechanisms underlying PF progression. CS exposure triggered pronounced fibrogenesis in the lung tissue and induced ferroptosis in the airway epithelium, consistent with previous reports using bleomycin‐induced PF models that highlighted the critical role of airway epithelial ferroptosis in PF pathogenesis.^[^ ^31^ ^]^ Our findings reveal the precise regulatory mechanisms of ferroptosis in the airway epithelium for therapeutic intervention.

The pathogenesis of PF begins with airway epithelial injury, which leads to impaired wound healing. Persistent inflammation, excessive ROS generation, and infiltration of activated immune cells such as macrophages, lymphocytes, and neutrophils exacerbate airway epithelial injury, compromising the respiratory barrier.^[^ ^32^ ^]^ To restore integrity, the surrounding fibroblasts are activated, leading to excessive ECM accumulation and dysregulated wound healing.^[^ ^33^ ^]^ Our previous study identified tPA as a mediator that links ferroptotic airway epithelium to fibroblast activation via the Glut1‐AMPK axis.^[^ ^17^ ^]^ Building on these findings, we discovered that mitophagy, the cellular process of removing damaged mitochondria, was inhibited in the ferroptotic airway epithelium of fibrotic lung tissues, owing to activated STAT6 signaling. Mechanistically, STAT6 translocated to the nucleus and inhibited PRKN transcription through binding to the site in its promoter, resulting in mitophagy inhibition. The resulting accumulation of dysfunctional mitochondria leads to increased ROS production and iron overload, further exacerbating ferroptosis.

Ferroptosis can be induced in the airway epithelium during the transition from ALI to PF, which may be a contentious aspect of the process.^[^ ^34^, ^35^ ^]^ In the early stages, airway epithelial ferroptosis is primarily driven by inflammation and ROS, making antioxidant therapies or targeting the SLC7A11‐GPX4 axis more effective.^[^ ^36^ ^]^ Our previous study demonstrated that STAT6 activation indirectly regulates SLC7A11 transcription to suppress lipid peroxidation and ferroptosis in ALI.^[^ ^18^ ^]^ Early interventions with antioxidants or anti‐inflammatory agents have shown promise in attenuating fibrogenesis.^[^ ^37^ ^]^ However, in later stages, therapies such as nintedanib and pirfenidone fail to halt disease progression.^[^ ^38^, ^39^, ^40^ ^]^ This highlights the complexity of persistent epithelial damage in advanced PF and underscores the need for novel therapeutic strategies. The current study indicates that restoring mitophagy to eliminate mitochondrial dysfunction could be a promising approach for mitigating epithelial ferroptosis and PF progression during these advanced stages.

STAT6 plays a dual role in lung injury and the progression of PF. Our previous study revealed that STAT6 mRNA and protein levels increase in the airway epithelium during the early stages of lung injury, driven by inflammatory cell infiltration and macrophage polarization.^[^ ^18^ ^]^ M2 macrophage‐secreted cytokines, including IL‐4, IL‐13, and TGF‐β1, activate STAT6, which initially protects against epithelial ferroptosis by enhancing SLC7A11 transcription via p53 inhibition. However, in advanced fibrosis, persistent STAT6 activation disrupts mitophagy balance, leading to mitochondrial dysfunction and ferroptosis. Our current findings demonstrate that sustained STAT6 activation suppresses PRKN transcription, resulting in inhibition of mitophagy, dysfunctional mitochondrial accumulation, and exacerbated epithelial ferroptosis during PF progression. Consistently, decreased PRKN has been reported in the lungs of actual PF patients, which further identifies the critical role of PARK2‐mediated mitophagy in PF pathogenesis.^[^ ^41^ ^]^

To investigate the regulatory roles of STAT6 and PRKN in vivo, we used mouse models with genetic modifications targeting STAT6 and PRKN. Given the complex composition of airway epithelial cells, no specific promoter is available for conditional regulation. To address this issue, we used WT and STAT6 KO mice to compare lung tissue fibrosis after CS exposure. Next, lentivirus‐mediated gene regulation was used to directly regulate STAT6 and PRKN expression via intratracheal instillation. By regulating STAT6 expression in WT and KO mice, we confirmed that STAT6 activation in the airway epithelium was positively associated with ferroptosis and mitochondrial dysfunction, and negatively regulated mitophagy. Silencing PRKN in WT and STAT6 KO mice further validated that STAT6‐mediated inhibition of mitophagy via PRKN suppression exacerbated ferroptosis and PF progression.

Given the therapeutic potential of STAT6, we screened suitable inhibitors. Currently, STAT6 inhibitors are rarely reported, and the available peptides are limited by their short duration of action.^[^ ^27^ ^]^ Most studies have focused on the inhibitors of STAT1, STAT3, and STAT5, leaving a gap in STAT6‐specific drug development.^[^ ^42^, ^43^, ^44^ ^]^ To address this, we performed a virtual screening of FDA‐approved compounds and identified rifabutin as a promising candidate. Rifabutin is a spiro‐piperidyl‐rifamycin that is structurally similar to rifampicin. It is currently used for the treatment of *Mycobacterium tuberculosis* and *Mycobacterium avium* in people infected with the human immunodeficiency virus.^[^ ^45^ ^]^ As an antibiotic, rifabutin is well‐tolerated and has a high safety profile. Compared with rifampicin, it is more permeable and accumulates more readily in cells.^[^ ^46^ ^]^ As an FDA‐approved drug, rifabutin has a well‐characterized pharmacokinetic and toxicological profile, which significantly reduces the risks associated with early‐phase clinical trials. Consistently, our experimental results demonstrated the remarkable protective effect of rifabutin on PF, with no significant harm observed in the lung tissue. Thus, rifabutin is an attractive candidate for the repurposing of PF treatment, bypassing the lengthy and expensive drug discovery process. These characteristics indicate that rifabutin is suitable for subsequent clinical trials as an antifibrotic drug. Additionally, owing to the increased risk of pulmonary tuberculosis in patients with PF, the use of rifabutin as an anti‐fibrosis agent may also effectively prevent the occurrence of pulmonary tuberculosis secondary to PF. Notably, the multifunctional effects of rifabutin make its direct application as a therapeutic agent for PF challenging, given the need to address off‐target effects and ensure safety. To address this, further studies, including in vivo evaluations and rigorous safety assessments, are warranted, particularly in the context of PF. These efforts will be critical to fully understand the feasibility of rifabutin and optimize its use for this new indication.

This work has certain limitations that should be considered. Although this study offers valuable insights into the role of STAT6 in PF and highlights rifabutin as a promising therapeutic candidate, certain aspects require further investigation. For instance, although we investigated the regulatory role of STAT6 in PRKN‐mediated mitophagy and ferroptosis in vivo, the inherent heterogeneity of airway epithelial cells could not be fully dissected due to current limitations in cell‐type‐specific promoter availability. To address this, we employed lentivirus‐mediated gene regulation via intratracheal instillation to modulate STAT6 and PRKN expression in airway epithelial cells. Additionally, the therapeutic efficacy of rifabutin has been evaluated primarily using in vitro and preclinical models, and its long‐term safety and off‐target effects in the context of PF require further investigation. Lastly, while this study focuses on the STAT6‐PRKN axis, other pathways contributing to PF pathogenesis remain to be explored. Future research addressing these aspects will strengthen the translational potential of our findings.

## 4 Conclusion

In summary, our findings reveal that STAT6 activation promoted PF by suppressing PRKN‐mediated mitophagy in the airway epithelium, leading to mitochondrial dysfunction and ferroptosis. Rifabutin was validated as a STAT6 inhibitor that effectively alleviates PF and offers a new therapeutic strategy. This study not only elucidates a novel STAT6‐PRKN regulatory axis in PF but also offers a lead compound for therapeutic development, paving the way for improved interventions in PF.

## 5 Experimental Section

### Animal Experiment

BALB/c and STAT6 knockout (KO) mice were procured from the Jackson Laboratory. Sex‐ and age‐matched BALB/c mice were used as wild‐type (WT) controls. The mice were maintained under a standard 12 h dark/light cycle with ad libitum access to water and food. Mouse handling in this study adhered to the Guide for the Care and Use of Laboratory Animals, and all procedures were supervised and approved by the Laboratory Animal Welfare and Ethics Committee of Chongqing University (CQU‐IACUC‐RE‐202402‐001).

The CS‐induced murine PF model was established as previously described.^[^ ^17^ ^]^ The CS particles were sourced from the U.S. Silica Company (Frederick, MD, USA). Briefly, mice were anesthetized via intraperitoneal injection of pentobarbital sodium and intratracheally instilled with a crystalline silica suspension (a 50 µL solution containing 3 mg particles) once, and sacrificed on day 56.

Lentivirus‐mediated short hairpin RNAs (shRNAs) targeting mouse STAT6 (target sequence: GGTTCAGATGCTTTCTGTTAC), PRKN (target sequence: GCTGGGACGATGTCTTAATTC), and the negative control lentivirus were intratracheally administered to mice. The specific and control viruses were designated as sh‐STAT6, sh‐PRKN, and sh‐NC, respectively. For the rescue assay, STAT6 KO mice were intratracheally administered lentivirus‐mediated STAT6 expression plasmid or a negative control lentivirus, named oe‐STAT6 and oe‐Vector, respectively. An equal amount of lentivirus was administered twice to the mice: one week before CS treatment and two weeks post‐CS administration.

For rifabutin intervention, mice from the control (CTRL) and CS groups were intraperitoneally injected with rifabutin (15 mg kg^−1^/day, MCE) until euthanized on the 56th day (*n* = 6 per group). The relevant biological samples were collected for subsequent analysis.

### Cell Culture and Treatments

Immortalized human bronchial epithelial (HBE) cells and human acute monocytic leukemia THP‐1 cells were purchased from ATCC (Manassas, VA, United States). HBE cells were cultured in Dulbecco's Modified Eagle Medium (DMEM) supplemented with 10% FBS (Hyclone) and 1% penicillin/streptomycin (Invitrogen). THP‐1 cells were grown in RPMI 1640 medium containing 10% FBS and 0.1% gentamycin (Invitrogen). The cells were incubated under a humidified condition of 5% CO_2_ atmosphere at 37 °C.

For CS stimulation, differentiated THP1 cells induced by 100 ng mL^−1^ phorbol‐12‐myristate‐13‐acetate (PMA, Sigma‐Aldrich) were initially exposed to CS (25 µg cm^−2^) for 72 h, and the conditioned medium (CM) was collected and used to treat HBE cells.

### Small Interfering RNA (siRNA) Transfection

Cells were transfected with the indicated siRNA using the HieffTrans in vitro siRNA Transfection Reagent (Cat No. 40806, YEASEN, Shanghai, China) according to the manufacturer's instructions. Non‐target siRNA, STAT6 siRNA, and Parkin siRNA were purchased from GenePharma with the target sequences: STAT6‐GCAGGAAGAACTCAAGTTT; Parkin‐GCCACGTGATTTGCTTAGA. Briefly, 20 pmol of siRNA and 12 µL of transfection reagent were mixed in 100 µL Optimedium (Invitrogen). After a 10 min room temperature incubation, the mixtures were added to the cells. Cells were used for subsequent studies after a 48 h incubation (37 °C, 5% CO_2_).

### Live‐Cell Imaging

HBE cells were transfected with mRFP‐GFP‐LC3 for 24 h and either left untreated or treated with rapamycin (Sigma‐Aldrich), bafilomycin A1 (BafA1, Sigma‐Aldrich), or CM. Subsequently, cells were gently washed with PBS, and visualization of mRFP‐GFP‐LC3 punctual formation was carried out using a fluorescence microscope (Olympus IX73, Japan).

### Histopathological Analysis, Immunohistochemistry (IHC) and Immunofluorescence Staining of Lung Tissue

Lung tissue samples were fixed in 4% paraformaldehyde and sectioned into 4 µm‐thick slices. Haematoxylin and eosin (H&E) and Sirius Red staining were performed as previously described.^[^ ^17^ ^]^ For IHC staining, the sections were incubated with primary antibodies overnight at 4 °C. Following PBS washing, the sections were incubated with HRP‐labeled Goat Anti‐Mouse/Rabbit IgG for 20 min at 37 °C. For IF staining, the sections were incubated with the indicated fluorescent secondary antibodies purchased from Yeasen (Fluor 488 anti‐mouse, 33906ES60; Alexa Fluor 594 anti‐mouse, 33212ES60; Alexa Fluor 488 anti‐rabbit antibodies, 33106ES60; Alexa Fluor 594 anti‐rabbit antibodies, 34212ES60) and DAPI (Solarbio, China, C0065) after washing off the primary antibody. Images were captured using a microscope (Olympus IX73, Japan).

### Indirect Immunofluorescence Staining

HBE cells were seeded onto glass coverslips (BS‐14‐RC, Biosharp Life Science, China). After fixing with chilled methanol for 15 min, the coverslips were incubated with separate primary antibodies and the respective secondary antibodies with DAPI for 50 min at room temperature. Stained cells were visualized under a microscope (Olympus IX73, Japan).

### Mitochondrial Function Assessment

MitoSOX and JC‐1 Staining were used to assess mitochondrial function. ROS in HBE cells was detected using the MitoSOX Red superoxide indicator (GLPBIO, GC68230) according to the manufacturer's instructions. The mitochondrial membrane potential in HBE cells was evaluated using JC‐1 Dye (Servicebio, C011‐2‐1) according to the manufacturer's guidelines.

### FeRhoNox‐1 Staining and Lipid Peroxidation Assessment

The labile iron content in HBE cells was assessed using FeRhoNox‐1, an Fe^2+^ fluorescent indicator (Maokangbio, China). Briefly, a working concentration of 5 µm FeRhoNox‐1 was utilized to treat HBE cells. After incubation at 37 °C for 60 min, cells were observed using the fluorescence microscope. Lipid peroxidation was measured using the C11 BODIPY 581/591 (GLPBIO, GC40165). Briefly, HBE cells were treated with C11 BODIPY 581/591 at a working concentration of 10 µm for 30 min in the dark, and then cells were observed using the fluorescence microscope (Olympus IX73, Japan).

### Dual‐Luciferase Reporter Assay

HBE cells were seeded in a 24‐wells plate and transfected with the PRKN promoter luciferase constructs along with thymidine kinase – Renilla luciferase (internal control, Promega) using PEI 40000 reagent (40816ES03, Yeasen, China). Luciferase activity was evaluated using the Dual Luciferase Reporter Gene Assay Kit (RG027, Beyotime, China) following the manufacturer's instructions.

### Chromatin Immunoprecipitation (ChIP) Assay

HBE cells were crosslinked with 1% formaldehyde at room temperature for 10 min, and the nuclear extracts were sonicated. After preclearing, the sonicated cell lysates were immunoprecipitated with the indicated antibodies along with protein A/G beads overnight at 4 °C. Reversion of cross‐linking was performed by heating the samples at 65 °C overnight, and the DNA was purified by phenol‐chloroform extraction. ChIP DNA amounts for the gene promoters of interest were analyzed using real‐time qPCR. The sequences of the primers used in this study are presented in Table S1 (Supporting Information).

### Transmission Electron Microscopy

Lung tissues were fixed in a solution containing 3% glutaraldehyde and 2% paraformaldehyde in 0.1 m cacodylate buffer at 4 °C. Following alcohol gradient dehydration, samples were embedded in Epon resin and sectioned using a Leica Ultracut microtome. Subsequently, sections were stained with uranyl acetate and lead citrate using a Leica EM Stainer. Electron micrographs were then analyzed using a Tecnai Spirit electron microscope.

### Immunoblot Analysis

Lung tissues and cells with indicated treatments were lysed with RIPA buffer containing protease and phosphatase inhibitors (FD1001, FD1002, Fudebio, Hangzhou, China), and the total protein content was quantified using a BCA protein assay kit (FD2001, Fudebio, Hangzhou, China). Equal amounts of protein from each group were separated by SDS‐PAGE, transferred onto a PVDF membrane (Millipore), and incubated with specific primary antibodies overnight at 4 °C. Primary antibodies against PTGS‐2 (sc‐376861), FTH‐1 (sc‐376594), STAT6 (sc‐374021), p‐STAT6 (sc‐136019), Parkin (sc‐32282), Pink1 (sc‐518052), LC3 (sc‐398822), Epcam (sc‐53532), β‐actin (sc‐47778) were purchased from Santa Cruz (Texas, USA). The 4‐HNE (bs‐6313R) was obtained from Bioss. A pre‐stained protein marker (Cat No. 20350; Yeasen, Shanghai, China) was used to identify the specific bands.

### Quantitative Real‐Time PCR Analysis (qRT‐PCR)

Total RNA was extracted from lung tissues or cells using an RNA extraction kit (M5101, NCM Biotech, China) following the manufacturer's instructions. RNA concentration was determined using a Nanodrop One (Thermo Scientific). Equal amounts of RNA were reverse transcribed using HiScript III All‐in‐one RT SuperMix Perfect for qPCR (R333‐01, Vazyme, China) in 8‐strip tubes (#403102, Nest, China). Real‐time PCR was performed using ChamQ Universal SYBR qPCR Master Mix (Q711‐02, Vazyme, China), and the relative expression of the gene was analyzed using the ΔΔCt method. The primer sequences used in this study are listed in Table S2 (Supporting Information).

### Seahorse Experiments

Oxygen consumption rates (OCR) were assessed using an Agilent Seahorse XF extracellular flux analyzer (Agilent Technologies) following a previously described method.^[^ ^17^ ^]^ For the OCR analysis, HBE cells subjected to the treatments were seeded in Seahorse assay plates and administered with Seahorse XF assay medium. After establishing the baseline OCR, measurements were taken following sequential injections of 5 µm oligomycin, 1.5 µm FCCP, and 5 µm rotenone.

### Microarray Data

All data were obtained from the National Center for Biotechnology Information (NCBI) Gene Expression Omnibus (GEO) (https://www.ncbi.nlm.nih.gov/geo/). Microarray‐based expression data of lung tissues treated with CS for 8 weeks were retrieved from GSE32147.^[^ ^47^ ^]^ Data of lung samples from WT and Stat6^−/−^ mice were obtained from GSE1438.^[^ ^48^ ^]^ Raw count data for normal human lung tissue samples were obtained from the Genome Tissue Expression (GTEx) database (https://www.gtexportal.org/home/).^[^ ^49^ ^]^

### Differentially Expressed Genes Analysis and Functional Enrichment Analysis

Differentially expressed genes (DEGs) were identified using the “limma” R packages with a significance threshold of *p* < 0.05. The R packages ggplot2 and Complex Heatmap were used to visualize the DEGs. Bioinformatic pathway analysis was conducted with Gene Set Enrichment Analysis (GSEA), considering only pathways with *p* < 0.05 and false discovery rate < 0.05 as statistically significant.

### Single Sample Gene Set Enrichment Analysis

Single‐sample GSEA (ssGSEA) was performed to calculate the enrichment score for PF and STAT6 signaling using the R package GSVA. This analysis applied gene signatures related to fibrosis and STAT6 signaling to each lung sample in the GTEx database.

### Spatial Transcriptome Analysis (STA)

Spatial transcriptomic data for crystalline silica‐induced PF in the murine model were acquired from the Sequence Read Archive (SRA) database under accession number SRP336178.^[^ ^50^ ^]^ The analysis was performed using the Space Ranger pipeline provided by 10X Genomics and R software. The R package Seurat 5.0.1 was utilized to normalize and visualize the dataset. Differentiation between normal regions and fibrotic lesions was established based on the expression of Col1a1, a classical fibrosis‐related gene. The presence of epithelial cells was identified using the cell marker Epcam, and further analysis was conducted to determine the involvement of STAT6‐regulated genes in epithelial cells using GSVA. Additionally, ferroptosis‐associated gene sets were downloaded from FerrDB, and the bioMart R package was used to convert homologous genes between humans and mice. Furthermore, gene ontology analysis was performed to compare lesions and normal areas, as well as to evaluate the difference in expression patterns between high and low involvement of STAT6‐regulated genes in epithelium.

### Molecular Docking and Visualization

The Protein Data Bank server was used to obtain the crystal structure of STAT6 and rifabutin‐BCL6 complex (PDB‐ID: 4Y5U, 4CP3, 1G5S).^[^ ^51^ ^]^ Furthermore, all solvents were removed from the protein structure, and hydrogen atoms were added to the protein using MGL Tools. The processed protein acts as a receptor. The ligand structure was downloaded from the PubChem database and converted to PDBQT format using MGL Tools batch preprocessing.^[^ ^52^, ^53^ ^]^ The 3D coordinates were generated using UNICON, for which only 2D structures were provided.^[^ ^54^ ^]^ Molecular docking calculations were conducted using Autodock Vina to obtain various possible orientations and binding energies for compounds at the active sites of the protein.^[^ ^53^ ^]^ Molecular docking analysis was carried out in DBD of STAT6 using coordinates: X = −11.933, Y = 24.597, and Z = −15.257 with dimensions of the grid box 82 × 108 × 66 Å. In addition, the parameters set in the coiled coil domain were X = −14.628, Y = −6.477, and Z = −40.386 with dimensions of the grid box 72 × 86 × 74 Å, and X = −10.548, Y = −7.359, Z = 15.159 with dimensions of the grid box 92 × 52 × 72 Å in the SH2 domain. Owing to the large volume of the grid box, the exhaustiveness was increased up to 20 to improve accuracy. The results of the molecular docking were then extracted, and the ligand conformation with the lowest binding energy was considered the best conformation. Finally, the LigPlot+ software was used to visualize the molecular interactions between the protein‐ligand complexes.^[^ ^55^ ^]^ To further verify molecular docking technique, the drug‐protein complex was taken apart, re‐docked the ligand, and the docked ligand was superimposed onto the original crystal structure.

### Statistical Analysis

The data were presented as the mean ± standard deviation (SD), as indicated in the figure legends. Unpaired Student's *t*‐test was used for comparisons between two groups. One‐way analysis of variance (ANOVA) was employed for comparisons between multiple groups with one variable, followed by Tukey's post hoc test. And two‐way ANOVA was performed to compare multiple groups with more than one variable. The sample size (*n*) for each experiment was stated in the figure legends. Statistical analyses were conducted using the GraphPad Prism software (version 9.0, GraphPad Software, CA, USA). Statistical significance was defined as a two‐sided *p*‐value of <0.05.

### Ethical Statement

Mice handling in this study adhered to the Guide for the Care and Use of Laboratory Animals, and all procedures were supervised and approved by the Laboratory Animal Welfare and Ethics Committee of Chongqing University (CQU‐IACUC‐RE‐202402‐001).

## Conflict of Interest

The authors declare that no conflict of interest.

## Author Contributions

Y.Y. performed methodology, and data curation, visualized and supervised the project, acquired funds, and wrote, reviewed & edited the final manuscript. G.H. performed data curation, visualized the project, and wrote the original draft. T.Z. performed methodology and data curation, and visualized the project, and wrote the original draft. Y.L. conceptualized the project, performed methodology and data curation, and designed the software. J.J. designed the software and visualized the project. D.D. conceptualized and supervised the project, investigated the data, and wrote, reviewed & edited the final manuscript. Y.M. performed methodology, data curation, visualized, and supervised the project. S.T. conceptualized, supervised, and visualized the project, acquired funds, and wrote, reviewed & edited the final manuscript.

## Supporting information



Supporting Information

## Data Availability

The data that support the findings of this study are available from the corresponding author upon reasonable request.
