# Supplementary material for: STAT6 Activation Exacerbates Ferroptosis in Airway Epithelium by Inhibiting PRKN‐Mediated Mitophagy in Pulmonary Fibrosis
Source: Adv Sci (Weinh). 2025 Jul 17;12(38):e01718. doi: 10.1002/advs.202501718 (PMC12520474; doi:10.1002/advs.202501718)
Supplement: Supplementary file 1 — Supporting Information [file ADVS-12-e01718-s001.docx]

**Supplementary Materials and Methods**

**1. Cell viability assay**

Cell viability was determined using the MTT assay. HBE cells were seeded at approximately 1×10^4^ cells per well in a 96-well plate. After indicated treatment for 48h, 20 μl MTT solution (2 mg/ml) was added for another 2 h incubation at 37 °C. Then, the supernatant was dislodged and 100 μl isopropanol/HCl was added. Absorbance at 570 nm was measured using a multifunctional microplate reader (Biotek Synergy H1).

**2. Detection of GSH, MDA and Iron content**

The content of GSH in HBE cells was detected using a commercial kit (A006-2-1, Nanjing Jiancheng Biotechnology, China). MDA production was quantified using the Lipid Peroxidation MDA Assay Kit (S0131, Beyotime, China) and the Iron Colorimetric Assay Kit (E-BC-K139-M, Elabscience, China) was utilized to measure iron content, following the respective manufacturer’s instructions.

**3. Protein and lactate dehydrogenase (LDH) content in BALF**

The concentration of protein and LDH in bronchoalveolar lavage fluid (BALF) was measured using commercial kits (FD2001,Fudebio, Hangzhou, China; A020–2-2, Nanjing Jiancheng Bioengineering Institute, China) according to the provided instructions.

**
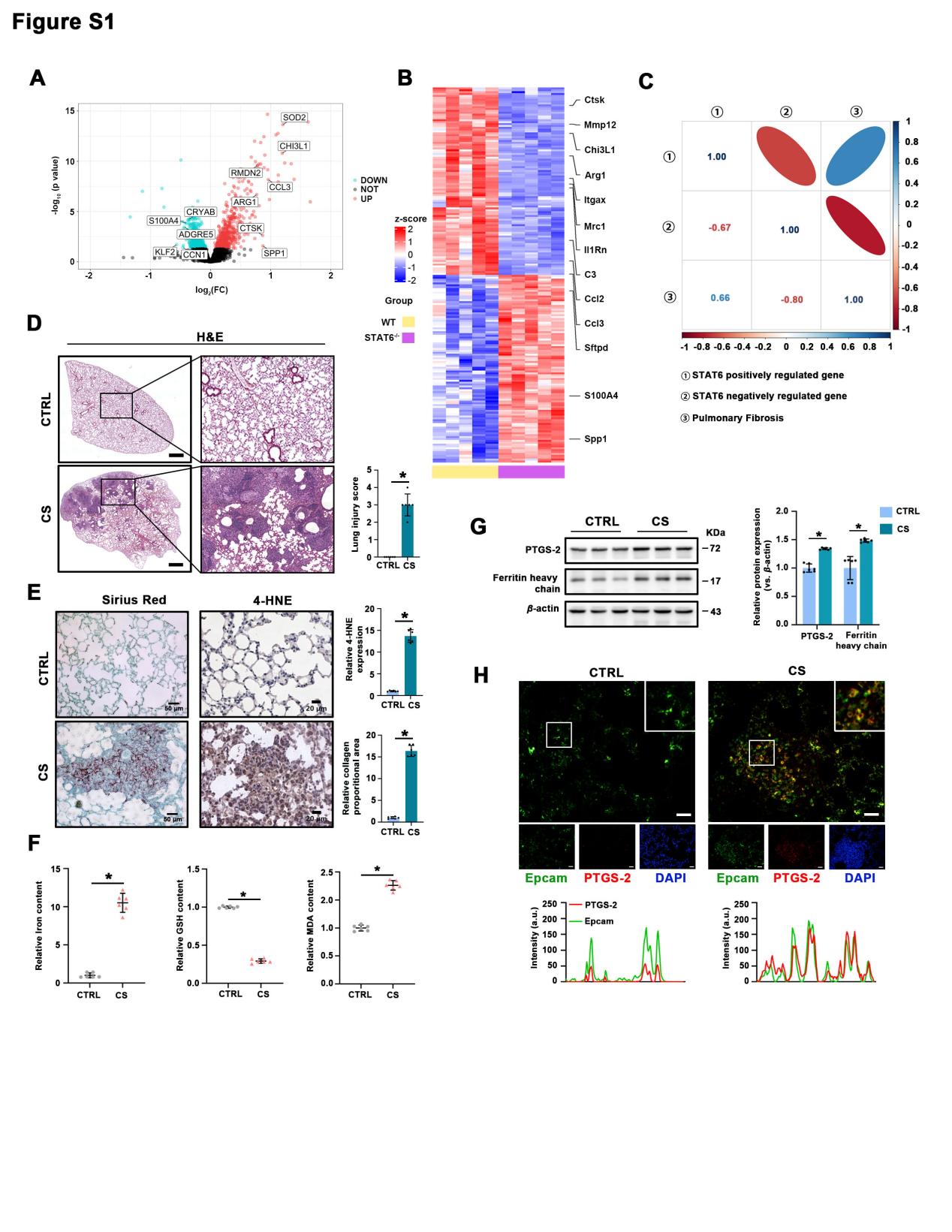

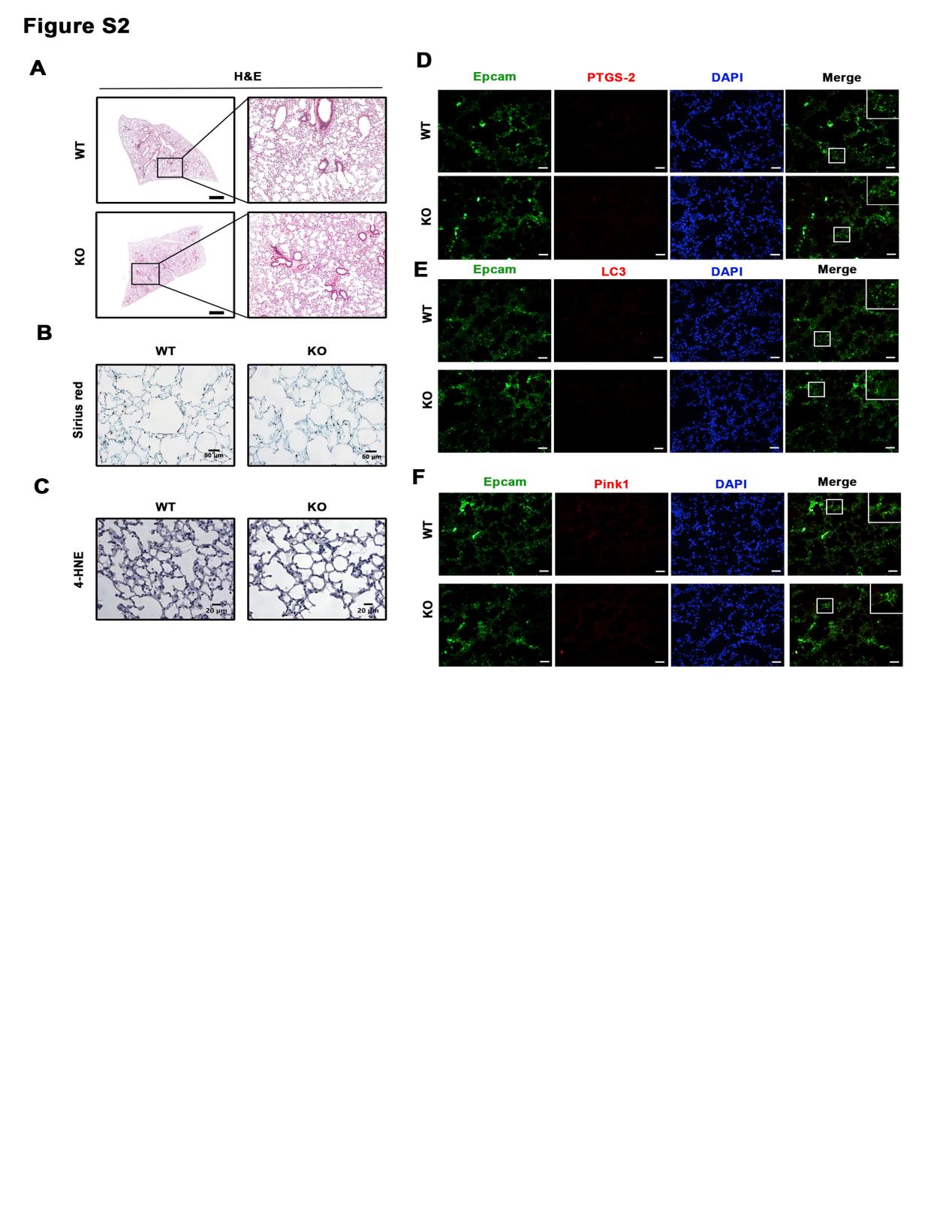

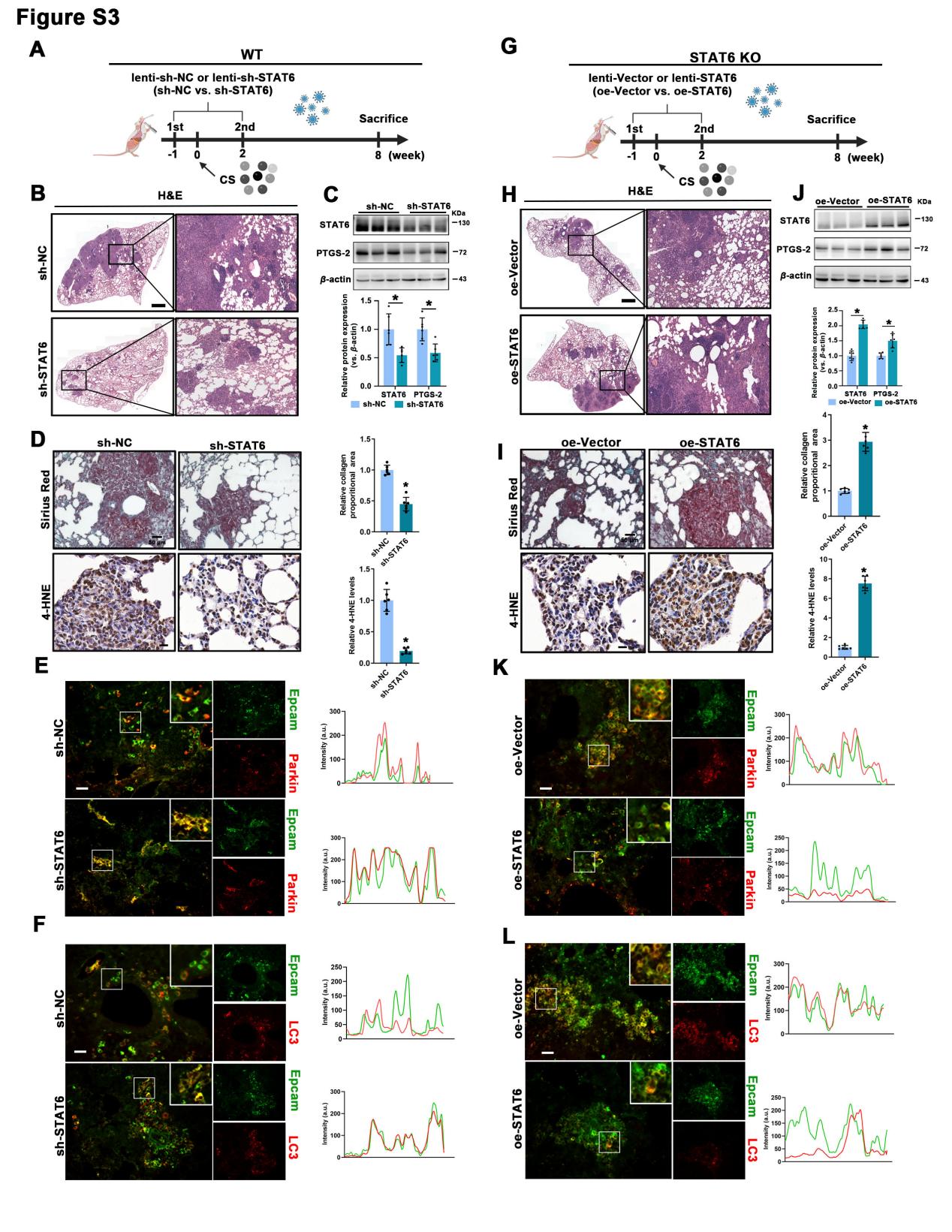

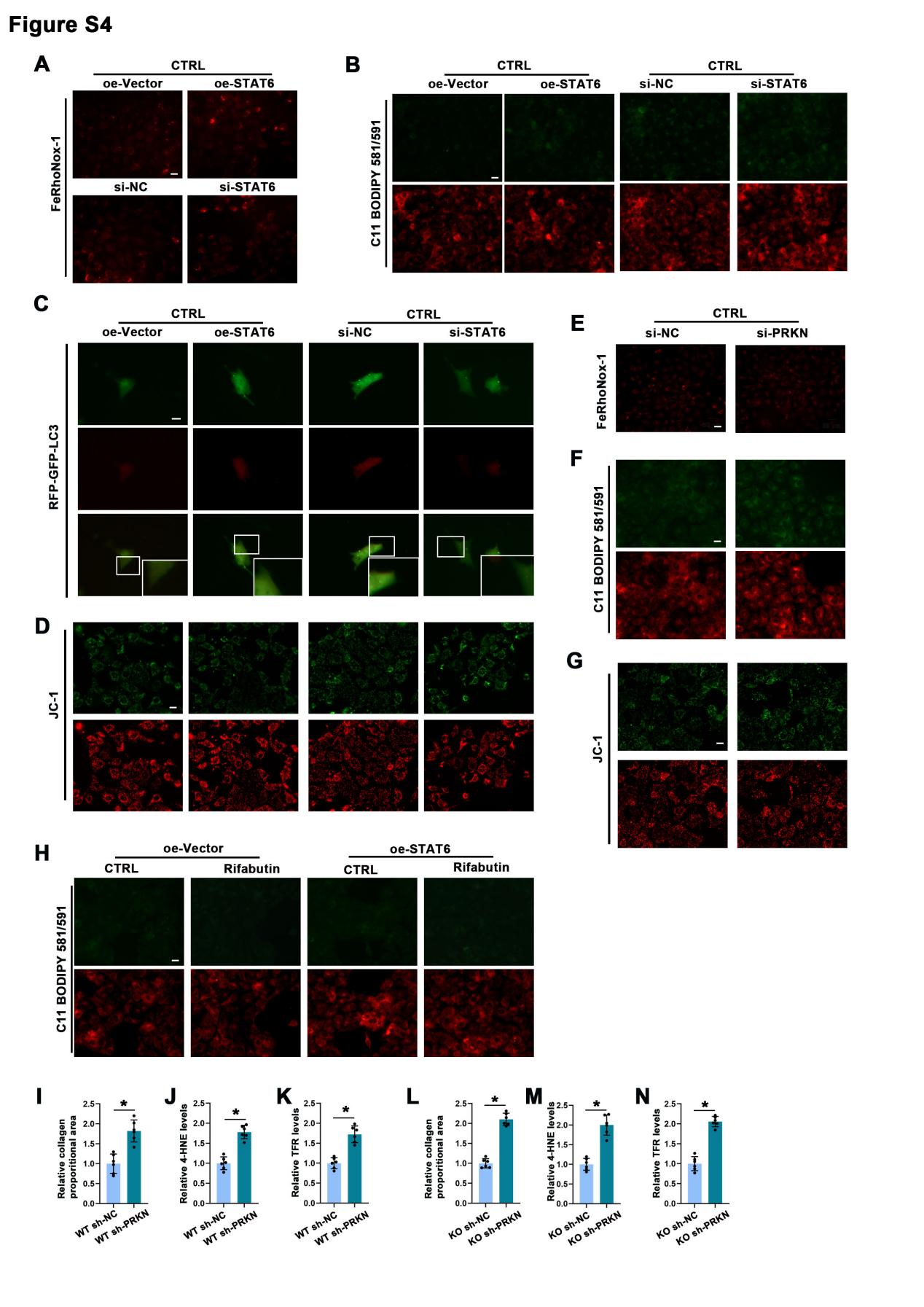

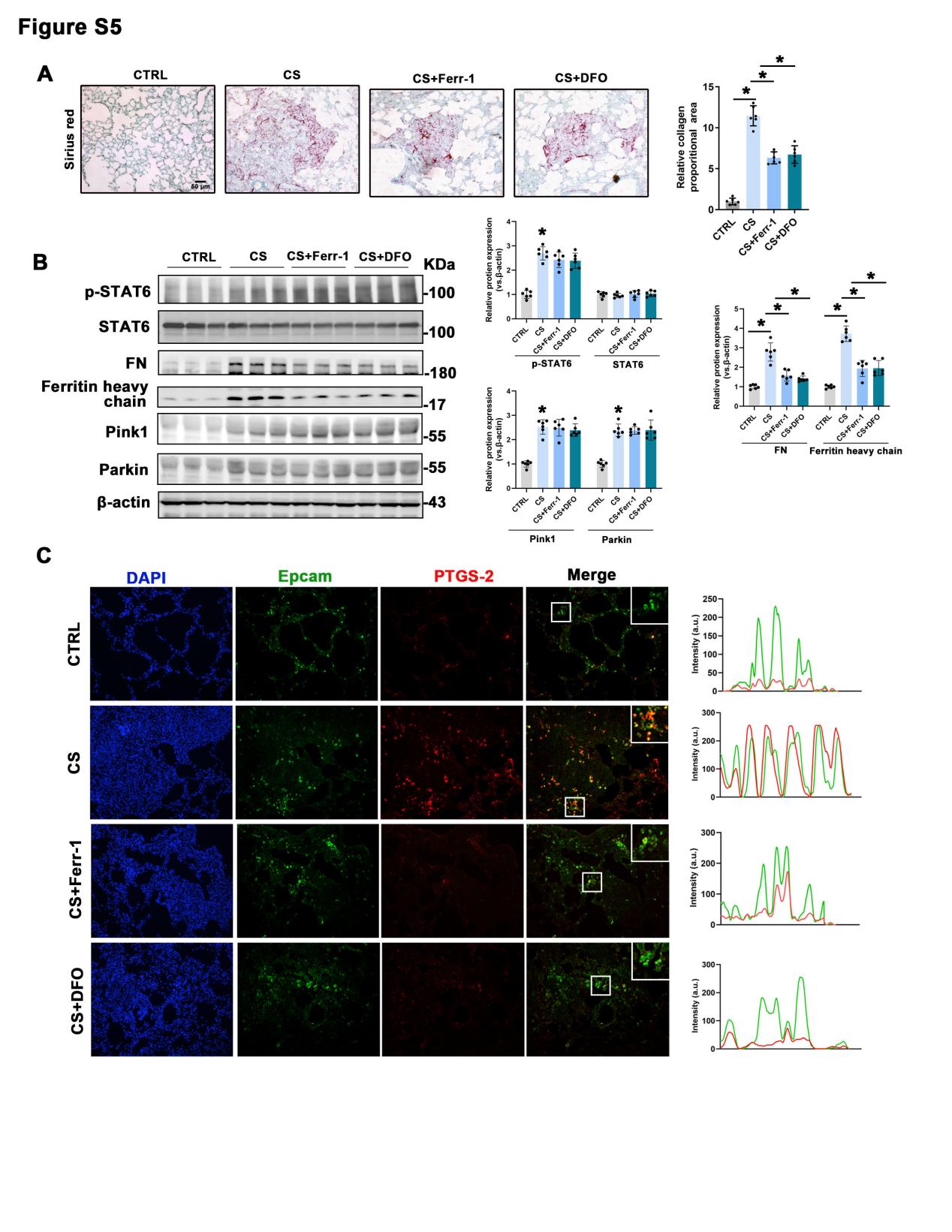

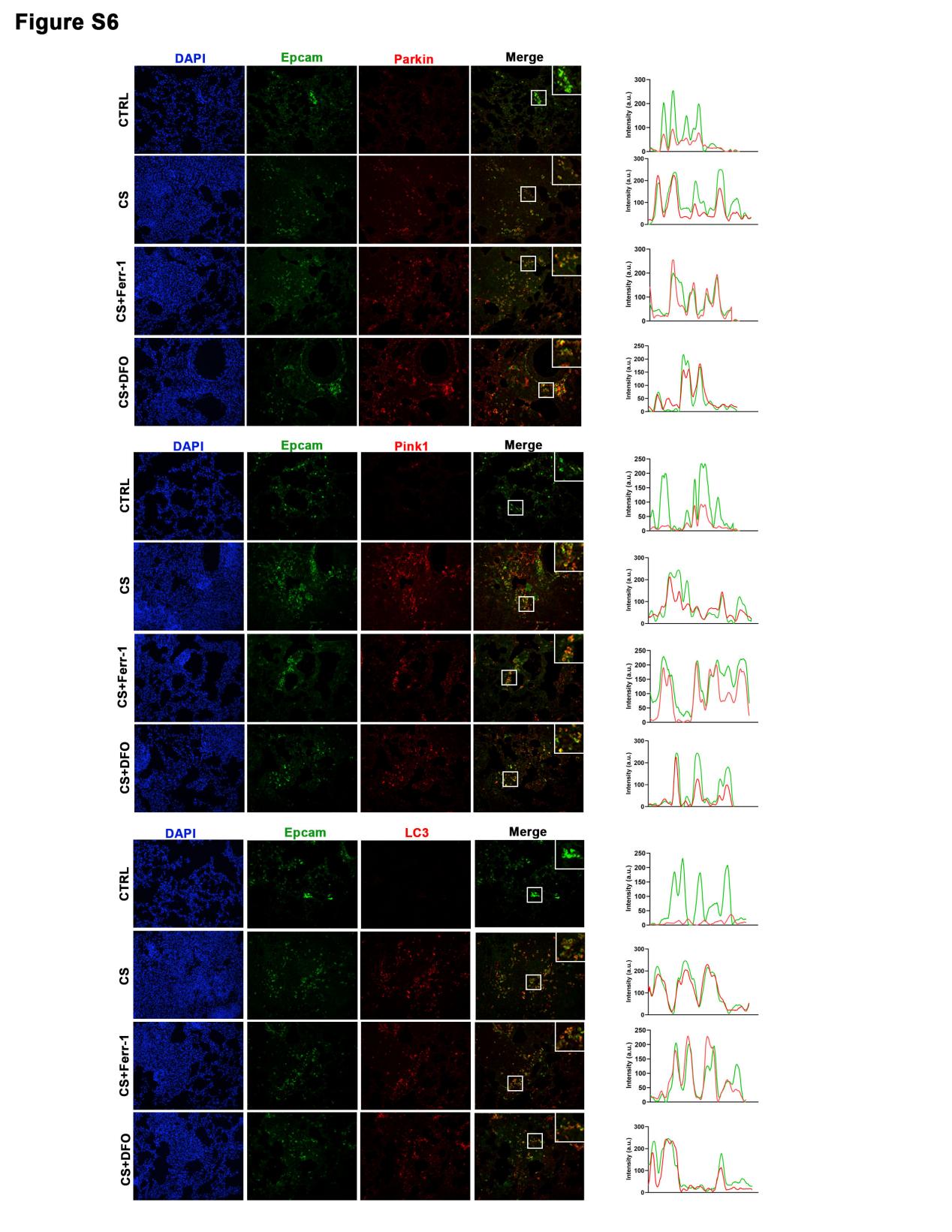

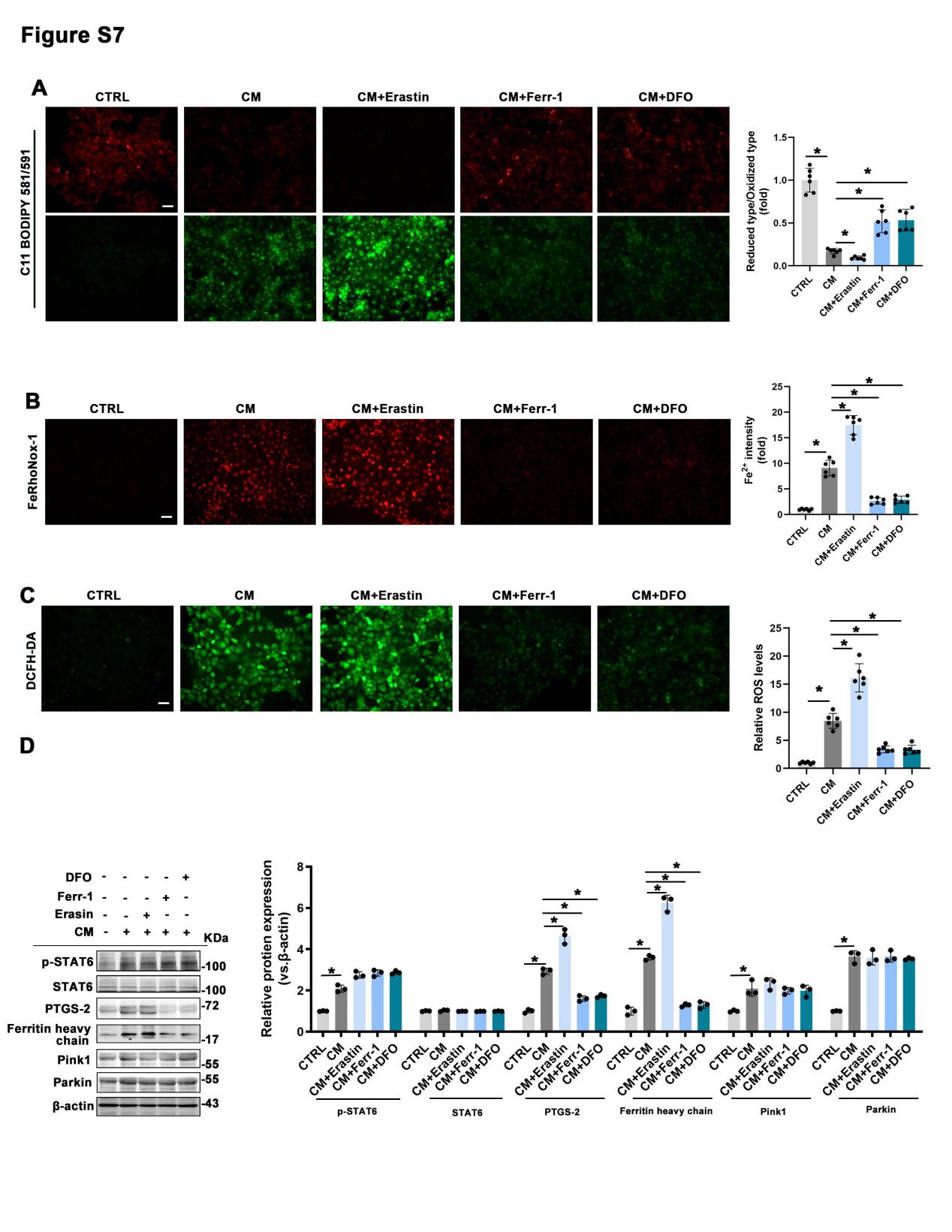

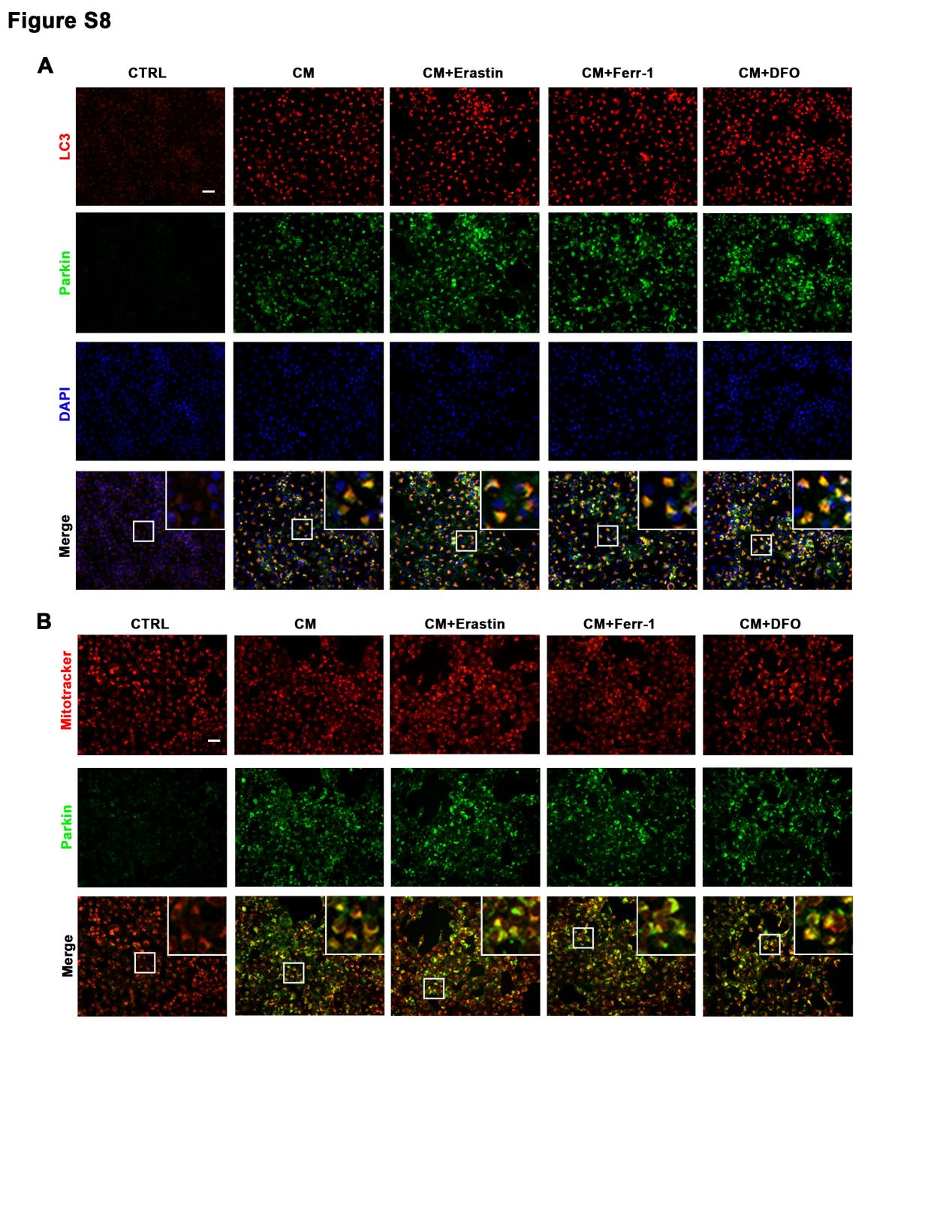
**

**Supplementary figure legend**

**Figure S1. STAT6 signaling pathway activation and ferroptosis jointly involves in CS-induced pulmonary fibrosis. (A)** Differential expressed genes in the CS-exposed murine model. **(B)** Heatmap showed genes negatively or positively regulated by STAT6 and pulmonary fibrosis-related genes were labeled. **(C)** The pearson correlation coefficient of GSVA score between STAT6 regulatory genes and pulmonary fibrosis-related genes. **(D)** Representative H&E-stained lung sections, scale bar: 1 mm. **(E)** Representative images of Sirius Red (scale bar: 50μm) and IHC (scale bar: 20 μm) staining (4-HNE) of lung tissue sections. **(F)** Relative Iron, GSH and MDA content in lung tissue were measured by corresponding kit. **(G)** The protein expression and quantification of PTGS-2 and ferritin heavy chain in lung tissue. **(H)** Representative IF co-staining of Epcam & PTGS-2 in lung tissue sections, scale bar: 50μm. The results were presented as means ± SD. *n* = 6 mice per group for D-H. Statistical analysis was performed using *t*-test for D-G. **p* < 0.05.

**Figure S2. STAT6 deficiency promotes airway epithelium mitophagy and suppresses ferroptosis in CS-induced lung injury. (A)** Representative H&E-stained lung sections from WT and STAT6 KO mice in the CTRL group, scale bar: 1 mm. **(B)** Representative images of Sirius Red staining of lung tissue sections in the CTRL group. **(C)** IHC staining of 4-HNE in lung tissue sections in the CTRL group were performed, scale bar: 20 μm. **(D)** Representative IF co-staining of Epcam & PTGS-2 in lung tissue sections of the CTRL group, scale bar: 50 μm. **(E)** Representative IF co-staining of Epcam & LC3 and Epcam & Pink1 **(F)** in lung tissue sections of the CTRL group, scale bar: 50 μm. *n* = 6 mice per group for A-F.

**Figure S3. Airway epithelium STAT6 negatively regulates mitophagy, which promotes ferroptosis and enhances pulmonary fibrosis. (A, G)** Schematic diagram of the lentivirus-mediated in vivo experiment. **(B, H)** Representative H&E- (scale bar: 1mm) and Sirius Red- (scale bar: 50 μm) stained lung sections. **(C, J)** The protein expression and quantification of PTGS-2 and STAT6 in lung tissue. **(D, I)** Representative images of IHC staining (4-HNE) of lung tissue sections, scale bar: 20 μm. **(E, K)** Representative IF co-staining of Epcam & Parkin in lung tissue sections in indicated group, scale bar: 50 μm. **(F, L)** Representative IF co-staining of Epcam & LC3 in lung tissue sections, scale bar: 50 μm. The results were presented as means ± SD. *n* = 6 mice per group for A-L. Statistical analysis was performed using *t*-test for C, D, J and I. **p* < 0.05.

**Figure S4.** **STAT6 mediates ferroptosis through negative regulation of mitophagy**. **(A-B)** Intracellular Fe^2+^ levels and Lipid ROS production in HBE cells with indicated groups were evaluated, scale bar: 20 μm. **(C)** HBE cells were transfected with a tandem mRFP-GFP-LC3 construct for 24 h and and with the indicated treatment. Representative image of autophagosomes (yellow puncta on overlay) and autolysosomes (RFP puncta on overlay), scale bar: 5 μm. **(D)** Mitochondrial membrane potential of HBE cells was detected by JC-1 staining, scale bar: 20 μm. **(E)** Intracellular Fe^2+^ levels, **(F)** Lipid ROS production and **(G)** Mitochondrial membrane potential in HBE cells with indicated treatments were evaluated, scale bar: 20 μm. **(H)** Lipid ROS production in HBE cells with indicated treatments were evaluated, scale bar: 20 μm. **(I, L)** Quantification of collagen in indicated groups. **(J, M)** Relative 4-HNE levels of indicated groups. **(K, N)** Relative TFR levels of indicated groups. *n* = 6 for A-N. Statistical analysis was performed using *t*-test for I-N. **p* < 0.05.

**Figure S5.** **Ferroptosis inhibitors significantly alleviate CS-induced PF with no effect on STAT6 signaling and mitophagy**. **(A)** Representative images of Sirius Red staining of lung tissue sections, and relative collagen quantification were showed in the right panel, scale bar 50 μm. **(B)** The protein expression of mice lung tissues were measured by immunoblot analysis and quantified. **(C)** Representative IF co-staining of Epcam & PTGS-2 in lung tissue sections, profile intensity showing their fluorescence signals, scale bar: 50μm. The data were presented as means ± SD. *n* = 6 mice per group for A-C. Statistical analysis was performed using one-way ANOVA with Tukey's post hoc test for A and B. **p* < 0.05.

**Figure S6.** **Ferroptosis inhibitors significantly alleviates CS-induced PF with no effect on mitophagy**. Representative IF co-staining of Epcam & Parkin, Pink1 and LC3 in lung tissue sections, profile intensity showing their fluorescence signals, scale bar: 50μm. *n* = 6 mice per group.

**Figure S7.** **The effect of regulation ferroptosis on CM-induced HBE cell damage, STAT6 signaling and mitophagy**. **(A)** Lipid ROS production in HBE cells with indicated treatments were evaluated and quantified, scale bar: 20 μm. **(B)** Intracellular Fe^2+^ levels in HBE cells with indicated treatments were evaluated and quantified, scale bar: 20 μm. **(C)** Intracellular ROS in HBE cells with indicated treatments were evaluated and quantified, scale bar: 20 μm. **(D)** The protein expression of HBE cells with indicated treatments were measured by immunoblot analysis and quantified. The data were presented as means ± SD. *n* = 6 for A-C, *n* = 3 for D. Statistical analysis was performed using one-way ANOVA with Tukey's post hoc test for A-D. **p* < 0.05.

**Figure S8.** **The effect of regulation ferroptosis on CM-induced HBE cell mitophagy.** **(A)** Representative IF co-staining of Parkin & LC3 in HBE cells with different treatments, scale bar: 50 μm. **(B)** Representative IF co-staining of Parkin & mitotracker in HBE cells with different treatments, scale bar: 50 μm. *n* = 6 for A-B.

**Supplementary Table 1: Primers for ChIP-PCR**

| **Species** | **Gene Name** | **Sequence (5'-3')** |
| --- | --- | --- |
| Human | Parkin#1 | Forward ACCTGAATGGTTATGGTATTGCTGA  Reverse TCTTGGAAATGGACTAAAATTCTGC |
| Human | Parkin#2 | Forward CAAGCAGAATTTTAGTCCATTTCCA  Reverse CCCATGTGGCTATGCAAAACG |
| Human | Parkin#3 | Forward CATGAGTTTATGTTCCCGGTTG  Reverse TAACTAGCCCCACTCAGCAAAG |
| Human | Parkin#4 | Forward GGTCCTCTTCGGCATCTTGT  Reverse ACCTCCTGCTCACATCCGTA |

**Supplementary Table 2: Primers for qRT-PCR**

| **Species** | **Gene Name** | **Sequence (5'-3')** |
| --- | --- | --- |
| Human | STAT6 | Forward GTCTGGTCTCCAAGATGCCC  Reverse ATATGCTCTCAAGGGTGCTGA |
| Human | PARKIN | Forward GACAGCAGGAAGGACTCACC  Reverse CCAGCAAGATGGACCCTGG |
| Human | GAPDH | Forward CTGACTTCAACAGCGACACC  Reverse TGCTGTAGCCAAATTCGTTGT |
| Mouse | STAT6 | Forward CTCTGTGGGGCCTAATTTCCA  Reverse CATCTGAACCGACCAGGAACT |
| Mouse | Arg-1 | Forward CGCCTTTCTCAAAAGGACAG  Reverse TTTTTCCAGCAGACCAGCTT |
| Mouse | Parkin | Forward ATGGGAGTCCAGGAGCTTGA  Reverse TTAACTGGACCTCTGGCTGC |
| Mouse | β-actin | Forward AAGGCCAACCGTGAAAAGAT  Reverse GTGGTACGACCAGAGGCATAC |

**Supplementary Table 3: Potential STAT6 binding site on Parkin promoter**

| **Score** | **Relative score** | **Start** | **End** | **Strand** | **Predicted site sequence** |
| --- | --- | --- | --- | --- | --- |
| 9.115482 | 0.864041941802033 | -990 | -976 | + | TGATTTCTAAGAACT |
| 8.466564 | 0.8551400438763156 | -470 | -456 | + | ATCTTCCTAGAAAAT |
| 6.1935625 | 0.8239588713341023 | -1824 | -1810 | + | CAATTTATGTGAAAT |
| 5.862855 | 0.819422205430427 | -1730 | -1716 | + | CACTTCCCAATAATT |

**Supplementary Table 4:** **Information of reference molecule and 10 hit compounds**

| **PubChem CID** | **Name** | **Structure^[[1]](#footnote-0)^** | **Affinity (kcal/mol)** | **Predictive *pIC_50_*** | **Category** |
| --- | --- | --- | --- | --- | --- |
| 24857889 | TMC-264 | 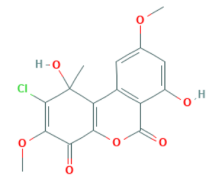 | -8.6 | 4.05 | Reference molecule |
| 3543 | 2-[trans-(4-Aminocyclohexyl)amino]-6-(benzyl-amino)-9-cyclopentylpurine | 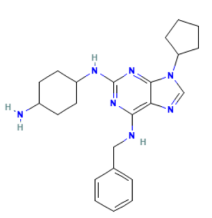 | -9.2 | 7.46 | Experimental |
| 4369241 | 6-Hydroxy-FAD | 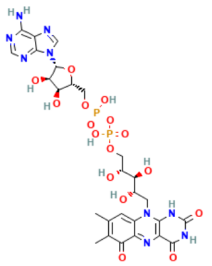 | -9.1 | 7.14 | Experimental |
| 444170 | Fenugreekine | 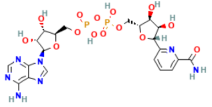 | -9.1 | 7.07 | Experimental |
| 135398743 | Rifabutin | 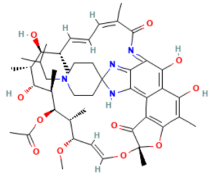 | -9.1 | 7.05 | Approved |
| 5289282 | 8-demethyl-8-dimethylamino-flavin-adenine-dinucleotide | 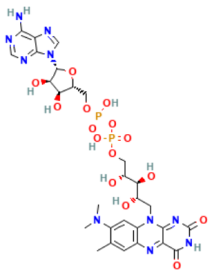 | -9 | 7.10 | Experimental |
| 643975 | Flavin adenine dinucleotide | 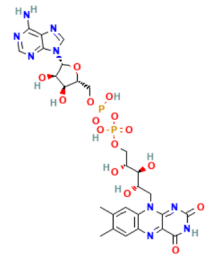 | -9 | 7.01 | Approved |
| 135451908 | N-[4-([(2-Amino-4-oxo-1,4-dihydropyrido[3,2-d]pyrimidin-6-yl)methyl]{(2E)-3-[4-carbamoyl-1-(5-O-phosphono-beta-D-ribofuranosyl)-1H-imidazol-5-yl]-2-propenoyl}amino)benzoyl]-L-glutamic acid | 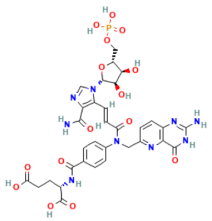 | -9 | 7.09 | Experimental |
| 6398457 | Flavin-N7 protonated-adenine dinucleotide | 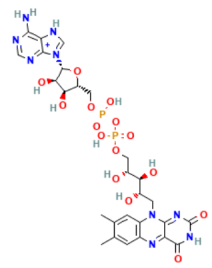 | -8.9 | 7.42 | Experimental |
| 170119 | Etheno-NAD | 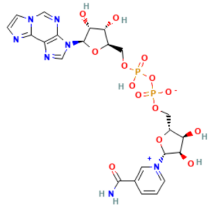 | -8.8 | 7.10 | Experimental |
| 16115747 | N-[2-(1,3-Benzodioxol-5-Yl)ethyl]-1-[2-(1h-Imidazol-1-Yl)-6-Methylpyrimidin-4-Yl]-D-Prolinamide | 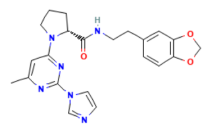 | -8.7 | 7.07 | Experimental |

1. ^1^ Structures were derived from screenshots of the PubChem website. [↑](#footnote-ref-0)
